# Supplementary material for: ‘Hybrid’ top down bottom up health system innovation in rural China: A qualitative analysis
Source: PLoS One. 2020 Oct 7;15(10):e0239307. doi: 10.1371/journal.pone.0239307 (PMC7540887; doi:10.1371/journal.pone.0239307)
Supplement: S1 Appendix — (DOCX) [file pone.0239307.s001.docx]

## S1 Appendix: The 22 Monitoring and Evaluation Indicators of HXI

| **Indicators** | **Definition** |
| --- | --- |
| 1．Ratio of hospitalization rate of MA (Medical Assistance scheme) vs. non-MA beneficiaries | Ratio of discharges per 100 MA beneficiaries and 100 non-MA beneficiaries |
| 2．Ratio of outpatient visit rate of the bottom 20% vs. the top 40% in the income distribution in the previous year | Households will be ordered according to per capita income, then bottom 20% and top 40% will be identified.  Ratio of outpatient visit rate of the bottom 20% vs. the top 40% in income distribution in the previous year |
| 3．% households with annual health expenditures in excess of 20% of total income | % of households with annual health expenditure in excess of 20% of annual total household income. |
| 4．Rate of overall satisfaction with rural health services among men | Rate of overall satisfaction with rural health services (outpatient and inpatient) delivered by all levels of health institutions among men, including satisfaction with time spent in hospitals, case explanation, equipment and infrastructure, bill inquiry, etc. |
| 5．Rate of overall satisfaction with rural health services among women | Rate of overall satisfaction with rural health services (outpatient and inpatient) delivered by all levels of health institutions among women, including satisfaction with time spent in hospitals, case explanation, equipment and infrastructure, bill inquiry, etc. |
| 6．Public health system scorecard rating | Criteria for scoring will be determined upon studies |
| 7．Innovation accepted and rolled out at provincial or regional level |  |
| 8．% of total annual (individual) inpatient expenses financed through out-of-pocket payments for NCMS members | % of total annual (individual) inpatient expenses financed through out-of-pocket payments for NCMS members. NCMS reimbursement or benefits by other health protection schemes are not part of out-of-pocket payment. |
| 9．% of total annual (individual) inpatient expenses financed through out-of-pocket payments for MA beneficiaries covered by the NCMS | % of total annual (individual) inpatient expenses financed through out-of-pocket payments for MA beneficiaries covered by NCMS. NCMS reimbursement and MA benefits are not part of out-of-pocket payment. |
| 10．Total annual NCMS expenditures as % of total annual NCMS funds | Total annual NCMS expenditures include expenditures on inpatient and outpatient reimbursement, check-up, risk reserve and other expenditures. Total annual NCMS funds refer to funds collected within the year, including budgetary allocation from central and local government, household contribution, government subsidy for target population, surplus of NCMS fund in the previous year, as well as other funds collected for NCMS. Funds are calculated upon actual number in the earmarked NCMS account. |
| 11．% NCMS enrollment rate | % of NCMS members in total agricultural population in the county |
| 12．Average number of outpatient visits per health professional per day over last year at THC | Average number of outpatient visits per health professional per day over last year, including outpatient and emergency services. |
| 13．Rate of change in average cost per inpatient case in county hospitals | Average expenditures per inpatient case in county hospitals |
| 14．% of deliveries by caesarean section | % of deliveries by caesarean section |
| 15．% of outpatients at township health centers and village clinics that receive two or more antibiotics | % of sampled prescription with two or more antibiotics in THCs and village clinics among all sampled prescriptions |
| 16．% of women who have access to a female qualified health worker | If required, women can have access to a female qualified health worker in THCs or village clinics. Information will be obtained through questionnaire targeting married women aging 15 to 49 |
| 17．% of villages that meet 'healthy village' standards | Criteria of healthy village will be determined upon studies. |
| 18．% of women between age 15 and 49 who undergo gynecological check-up in the last year | % of women between age 15 and 49 who undergo gynecological check-up in the last year gynecological check-up is limited to married women, not including disease-incurred or pregnancy check-up in hospitals. |
| 19．% of individuals over 35 who undergo hypertension screening in the three months | % of individuals over 35 who have blood pressure measured in the three months. Hypertension patients can be identified through blood pressure measurement and health check-up. Blood pressure measurement in this indicator includes measurement by health professionals and self-measurement. |
| 19．% of individuals over 35 who undergo hypertension screening in the three months | % of individuals over 35 who have blood pressure measured in the three months. Hypertension patients can be identified through blood pressure measurement and health check-up. Blood pressure measurement in this indicator includes measurement by health professionals and self-measurement. |
| 20．Annual per capita government expenditures on county level public health institutions and programs | Annual per capita government expenditures on county level public health institutions and programs refer to budgetary allocation at the county level on public health institutions and programs, as well as various earmarked funding in the field of public health. Public health institutions include CDC, MCH institutions, health personnel training institutions, health inspection institutions and other health institutions |
| 21．Project lessons and experiences are documented and disseminated (Qualitative) | No Measure Defined |
| 22．Project experiences are extended or adopted outside project areas (Qualitative) | No Measure Defined |
