# Supplementary material for: ‘Hybrid’ top down bottom up health system innovation in rural China: A qualitative analysis
Source: PLoS One. 2020 Oct 7;15(10):e0239307. doi: 10.1371/journal.pone.0239307 (PMC7540887; doi:10.1371/journal.pone.0239307)
Supplement: S2 Appendix — (DOCX) [file pone.0239307.s002.docx]

Shanxi Wuxiang

Zezhou

Yushe

Taigu

Heilongjian Gannan

Lindian

Fujin

Linkou

Jiangsu Gaochun

Liyang

Haimen

Gaoyou

Danyang

Jiangyan

Henan Yiyang

Ruzhou

Wuzhi

Qingfeng

Xi

Chongqing Jiulongpu

Qianjiang

Yongchuan

Rongchang

Liangping

Shizhu

Shaanxi Mei

Xunyi

Ningqiang

Hanbin

Zhenan

Gansu Gaolan

Huining

Gangu

Jingning

Kangle

Qinghai Datong

Huangzhong

Huangyuan

Ledu

Huzhu
