# Supplementary material for: ‘Hybrid’ top down bottom up health system innovation in rural China: A qualitative analysis
Source: PLoS One. 2020 Oct 7;15(10):e0239307. doi: 10.1371/journal.pone.0239307 (PMC7540887; doi:10.1371/journal.pone.0239307)
Supplement: S4 Appendix — (DOCX) [file pone.0239307.s004.docx]

# S4 Appendix: Interview Protocol – Extensive Topic List

## Chapter 1: General Topics

1. Key characteristics of intervention
2. Context specific and selection mechanisms
3. Adaptation mechanisms
4. Implementation processes and adjustments
5. Evaluation processes
6. Sustainability
7. Scale up and dissemination
8. Top-down and bottom-up changes
9. Processes and Tools for design and adaptation
10. Role of support by experts and project staff
11. Use of incentive mechanisms
12. Leadership

Topics 1-7 and 12 are elaborate via specific questions below. Topics 8-11 are approached via an initial open question (for instance, did you experience the reform to happen top down? and then later, did you experience the reform to be bottom up?), and follow up questions (can you elaborate, can you give an example,….)

Subsequent questions firstly follow the stage model of innovation proposed by Greenhalgh [10] and evidence based factors per stage. Not all questions need to be asked, they are topics that can be explored depending on the respondent and the interests of the respondents. Take ample time for the interview and have an open conversation.

## Chapter 1 Awareness and rationale

1. What was the problem(s) or issues your reform was attempting to address? *(1)*
2. How did you know this was a problem? Was there a formal assessment or survey? *(2)*
3. What were the long and short term objectives? *(5)*
4. What were the (intermediate and final) indicators used to benchmark or measure performance? *(9)*

## Chapter 2 Selection

1. Describe the/a selected intervention(s).
2. What were arguments and factors that influenced the selection of the innovation? (theory, evidence, experience,….)

## Chapter 3 Adaptation

1. What was the design? *(7)*
2. Provide detailed description on the design and the planning process. *(7)*
3. How were the sites (THCs, VCs, hospitals, villages, etc.) selected? *(7)*
4. What tools and instruments were used to prepare the design? *(8)*
5. Describe the iterative adjustments that were made during the implementation phase. Why were these adjustments made? *(12)*

## Chapter 4a Implementation (innovation itself)

1. What were the pathways to preparing the way for implementation (e.g., training, negotiations, policy environment, leadership, etc.)? *(10)*
2. How was the innovation implemented? Were there implementation phases? *(11)*
3. Relatively speaking, which innovation brought the most advantage? 相对而言，这个创新给我们带来的最大优势
4. Were the innovations compatible with your existing ways of working? 该创新与我们现有的工作方式没有冲突
5. This innovation had low complexity? 该创新复杂性最低
6. It was an innovation we could easily try out? 该创新最容易试点
7. The effects were clearly observable? 效果最易于观察
8. The innovation lend itself well for adaptation? 便于因地制宜的实施
9. It was a low risk innovation? 是个低风险创新
10. It was an innovation for which there was good technical support? 有良好的技术支持创新

## Chapter 4b Implementation (adopter) (Greenhalgh et al. 2005)

1. The intervention addressed our most important need? 干预措施满足了我们最为重要的需求
2. We were highly motivated when we started the implementation? 当我们开始实施创新措施时，我们有巨大的动力
3. Implementation of the intervention was expected to contribute to our most important goals? 望创新措施的实施有助于我们实现最为重要的目标
4. We had the skills required to implement the intervention ourselves or via our supporting partners? 我们有自己实施创新措施或和合作伙伴一起实施的必要能力
5. We are used to trying out and experimenting with innovations? 我们习惯对创新进行尝试实验的工作方式是习惯的
6. We exchange experiences with innovations regularly? 我们定期交流创新经验

## Chapter 4c Implementation (process)

1. We had the assistance of support teams? 我们有辅助团队的支持
2. Our leaders and managers provided practical help? 我们的领导提供实际帮助
3. We were well trained and hired extra staff where necessary? 我们接受了良好培训，必要时会招聘额外的工作人员
4. We had dedicated resources such as money, computers, facilities? 配备必要的资源，如财力，计算机及其他设备
5. Internal communication was well organized? 内部沟通顺畅
6. External communication was well organized? 组织有效的外部沟通
7. We had feedback on the process as we implemented? 实施过程中获得实施情况反馈实施

## Chapter 5 Evaluation

1. What were the long and short term objectives expressed in the same indicators whenever possible? *(5)*
2. Could you present the baseline data on the relevant indicators as previously indicated in one or more tables. *(3)*
3. What issues emerged with the measurement and analysis of indicators? Did the indicators change during implementation? If so, why? *(13)*
4. Did the innovation achieve the objectives as expressed in the indicators mentioned under 3 and 5? *(15)*
5. Do you think there were any side effects (on other indicators)? Provide the relevant data. Propose plausible explanations. *(16)*
6. What inferences/hypotheses/mechanisms do you see regarding the relationship between the effect on the one hand and the context and intervention on the other hand


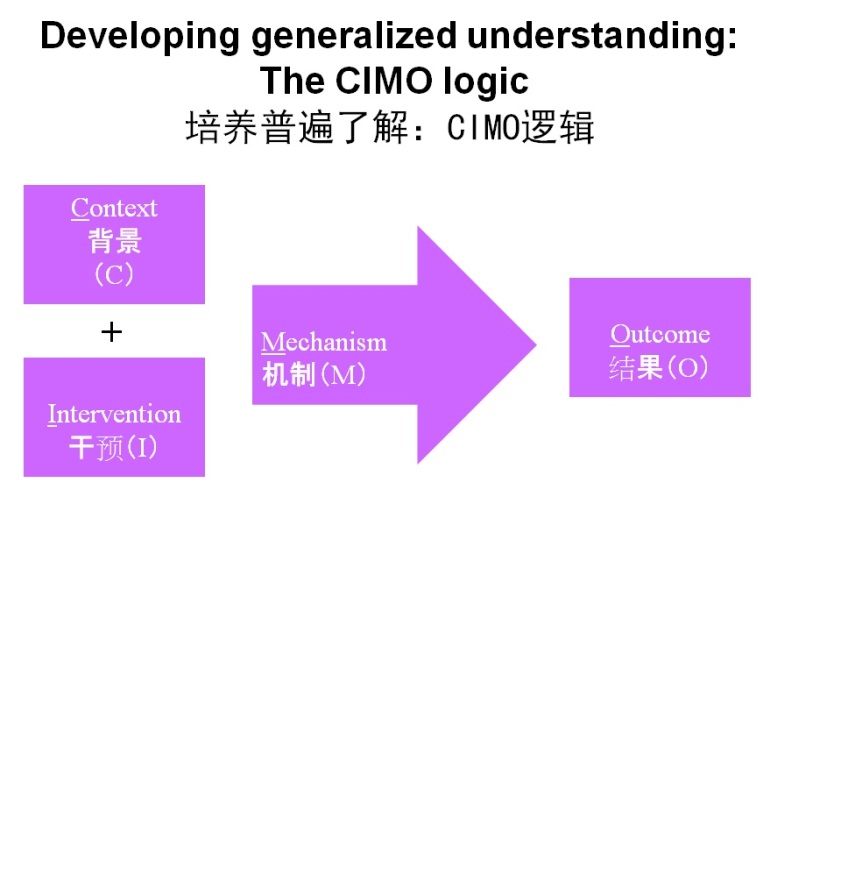


## Chapter 6 Context

1. How is/are the innovation(s) related to the National Rural Health Reform?
2. How do(es) the innovations relate to the provincial priorities (Health XI or other)?
3. Which non health-sector partners are involved (bureau of civil affairs, major, CPC,....)
4. Which health organisations are involved (NCMS office, Regional Health Bureau County Hospital. THC, Village Clinic, et cetera)
5. Which related innovations were taking place at the same time in the county?
6. How did the innovation(s) benefit from interaction via Health XI activities?
7. How was the leadership team composed?
8. Were there changes in the context on the above items? If so, please describe.

## Chapter 7 Sustainability

- Innovation characteristics 创新特征
  - Fit 适合
  - Ability to be modified/ modifications made能够被调整/进行的调整
  - Effectiveness or benefit有效性或效益
  - Ability to maintain fidelity/integrity能保持忠实性/完整性
- Context背景
  - Climate 气候
  - Culture 文化
  - Leadership 领导力
  - Setting characteristics (structure;policies)环境特征（结构；政策）
  - System/policy change体系/政策变化
- Capacity 能力
  - Champions (internal or external)拥护者（内部或外部）
  - Funding 资金
  - Workforce (staffing, attributes) 人力（人员、特点）
  - Resources 资源
  - Community/stakeholder support/involvement社区/利益相关者的支持/参与
- Processes and interactions过程与互动
  - Engagement/relationship building Shared decision making among stakeholders参与/关系建立利益相关者的共同决策
  - Adaptation/alignment 适应/一致性
  - Integration of rules/policies规则/政策整合
  - Evaluation and feedback评估与反馈
  - Training and education 培训与教育
  - Collaboration/partnership合作/合伙
  - Navigating competing demands 不同需求的通盘考虑
  - Ongoing support持续的支持

## Chapter 8 Dissemination (Slaghuis et al. 2013)

三因素模式的内容特征

- 1. Effectiveness of spread of work practices 传播工作做法的有效性
  2. In our organization / in other departments or teams: 规模：新工作方法的传播

在我们组织/其他部门/其他团队

- 1. Practitioners also use the documentation on the new work practice工作人员同样利用有关新工作方法的资料
  2. The required skills have been trained in other teams其他团队接受了必要技能的培训
  3. By now they have also developed the new, care-specific knowledge and skills required for the work practice 现在他们已经掌握了开展有关工作所需的新的知识和技能
  4. the practitioners have been instructed how to use the materials for the work practice工作人员已经学习如何利用相关资料用新的方法工作
  5. the practitioners now use the same, new materials for the work practice工作人员都使用新资料用新的方法工作
  6. Effectiveness of spread of results 传播结果的有效性
  7. Other institutions or counties strive for results like ours. 其他团队或部门力图取得同样的结果
  8. Other institutions or counties achieve similar results in quality of care其他团队或部门达到了同等的服务质量
  9. Other results have set a standard in new improvement projects in other institutions or counties我们的结果成为其他团队或部门新改进项目的标杆
  10. Professionals in other institutions or counties use the documentation and information that is available on our results其他部门或团队的专业人员使用我们项目结果的文件和信息
  11. In sum, to what extend have the changes in your care practices, as developed and implemented by your improvement team, been spread to other institutions or counties? 我们改进团队设计及实施的工作改进方式在多大程度上被传播到其他部门或团队
  12. To what extent have the changes been implemented effectively in other institutions or counties? 改进措施多大程度上在你们组织的其他部门或团队得到了实施

Actions for spread of results 传播结果

1. The results of the work have been made public: 对外公布解新的工作方法所取得的成效
   1. In presentations (in discussion of progress, formal meetings or other occasions) 会议发言（进展讨论或其他场合）
   2. in the annual reports or other reports年报或其他进展报告
   3. informally in discussions of progress or consultations关于项目进展的非正式交流
   4. in refresher courses or activities 培训课程或活动中

Actions for spread of work practices 传播工作做法

1. to spread the work practice we have: 就通过下面的方式介绍的新工作方法进行推广
   1. presented the work practice in discussions of progress在进展讨论会中介绍新工作方法
   2. organized clinical trainings, workshops and other refresher courses临床培训，研讨会或其他培训
   3. organized guest visits and exchange for professionals in the other teams /departments. 参观，与其他部门或团队的人员进行互访

## Chapter 9 Leadership (Ovretveit 2005)

1. How well prepared were you (“readiness for change”)
2. How did you formulate your vision and strategy (participatory)
3. Where there any Structural and line management process changes
4. What systems changes did you implement for/as leaders (data collection and reporting)
5. Did you implement supporting interventions on Human resources, people and team development
6. How did you approach Communication, commitment and motivation
7. Did you lead by example? Actions speak louder than words
8. What was your assessment of the initial situation (type, priority, extern-intern)
9. How did you assess your influence (more and less)
10. Did you encourage adaptation instead of adoption, i.e. Translate, don’t transfer (no copy paste)
11. Was it difficult to balance between areas 1 to 7 above?

## Miscellaneous Pilot county focus items

Scan for topics below that you may feel deserve further attention still. If so, ask open questions.

- performance review practice
- system of hospital points to qualify the hospital for a variable bonus amount
- Top down vs. bottom up implementation
- benchmarking performance-based management and effects experienced
- operation guidelines
- translate or transfer
- capacity building
- positive and negative incentives
- hospital autonomy (internal – external evaluation)
- performance-based management: adverse effects
- used indicators: change and weight
- transparancy and communication of indicators and weight
- role of IT
- relative effect and effect
- continuation after HXI
