# Supplementary material for: ‘Hybrid’ top down bottom up health system innovation in rural China: A qualitative analysis
Source: PLoS One. 2020 Oct 7;15(10):e0239307. doi: 10.1371/journal.pone.0239307 (PMC7540887; doi:10.1371/journal.pone.0239307)
Supplement: S5 Appendix — (DOCX) [file pone.0239307.s005.docx]

**S5 Appendix. CPSM and World Bank Project reports**

1. Innovation & Development, Mid-term Progress and achievements of China Rural Health Care Project. CPSM, September 2011.
2. China Rural Health Project, Indigenous Peoples Planning Framework (IPPF),
3. Effect evaluation Draft Reports on the Comprehensive Reform of county-level medical institutions
4. Report about Mid-Term Review for China Rural Health Project
5. China Rural Health Project Mid-Term Review Report (Draft for Discussion)
6. Effect Evaluation Reports on the Comprehensive Reform of County-level Medical Institutions
7. Innovative Approaches to the Financing and Provision of Public Health Services in Henan Province
8. Spot Assessment of the Performance Evaluation Pilot Project in 5 Counties in Rural China
9. International Technical Assistance for Workshop on Applying and Promoting Evaluation Index on Public Health System in China’s Rural Health Project Supported by World Bank Loan/UK Grant
10. The Development and Application of Scoring Indicators for Public Health System
11. The Evaluation Index System for Healthy Village and It’s Pilot study
12. Change Mechanism, Control Cost and Ensure Quality to Benefit the People, Enlightenment from the comprehensive pilot reform of county hospital
13. The evaluation criteria for the base salary and 70% performance-based salary in 2014
14. The evaluation criteria for the monthly bonus as 30% of salary in 2014
15. Presentation: construction of the new mechanism of the two-grade performance evaluation
16. The implementation guideline of the performance evaluation for township hospitals in Mei county, March 2011
17. Presentation: the two-grade (township and village level) performance evaluation, including the introduction, design and assessment, May 2014
18. Implementation guideline of the performance evaluation, 2014, Majia hospital, Mei county
19. Implementation guideline of the quality assessment of the health service and the performance evaluation for the village level clinic in Mei county,, September 6, 2010
20. Implementation guideline of the performance evaluation of the basic public health service in Mei county, 2014
21. Implementation guideline of the performance evaluation of the township and village level hospital in Mei county, September 4, 2009
22. Annual plan of Xi county (2009-2014) the annual plans of Xi county from 2009 to 2014, including the plan of the funds as incentive in 2012
23. Presentation: the report of the integrated payment reform in Xi county, May 26, 2014
24. Presentation: the report of the progress of the integrated payment reform and coordinated health service in Xi county, May, 28, 2014
25. Presentation: the report of Xiazhuang town, including the intergrated payment reform, the coordinated health service and the basic public health service, May, 26, 2014
26. Notification of the integrated payment reform (i.e. surveillance of the quality of the health service), by Xi county People's Hospital, 2013
27. Notification of the adjustment of the integrated payment reform (i.e. surveillance of the quality of the health service) and performance evaluation, by Xi county People's Hospital, 2014
28. Technical report of the cost estimation for basic public health service package
29. Technical report of the manpower hours estimation of the nationwide basic public health service package (taking Jiulongpo county, Chongqing for example)
30. Report of the piloting work of H11 in the central hospital of Tongguanyi town
31. Implementation guideline of the performance evaluation for the basic level hospitals in Jiulongpo
32. Guideline of the performance evaluation for the public health and basic health service in Jiulongpo, Chongqing. October 10, 2011
33. Evaluation criteria for the performance evaluation for the basic level health care organisations in Jiulongpo, version 2013
34. Implementation guideline issued by the government of Jiulongpo or the health bureau of Jiulongpo, e.g. the implementation guideline of accelerating the development of the rural and urban community health service; guideline for management of the rural and urban basic public health service
35. Manuscript by Jiulongpo CDC and health bureau; the title is: Research on the Human Cost of the National Public Health Service Package of Chongqing
