# Supplementary material for: ‘Hybrid’ top down bottom up health system innovation in rural China: A qualitative analysis
Source: PLoS One. 2020 Oct 7;15(10):e0239307. doi: 10.1371/journal.pone.0239307 (PMC7540887; doi:10.1371/journal.pone.0239307)
Supplement: S6 Appendix — (DOCX) [file pone.0239307.s006.docx]

**S6 Appendix: Case Study Descriptions**

**Box 1: Case study 1: Comprehensive Hospital Payment Reform in Xi Xian, Henan Province**

**County characteristics**

Xi Xian County is located in Henan Province, in Central-Eastern China. It is under the administration of Xinyang city, covers 1,836 km^2^ and has a population of 793,746. With a per capita GPP of around 34.000 RMB, the Henan province is significantly below the Chinese average.

**Baseline assessment**

The innovation was designed to address variation in reimbursement rates, high unexpected out-of-pocket costs, and related patient dissatisfaction, as already on the county and provincial agenda for some time and common across rural China. It related to the HXI objectives on hospital payment and service delivery improvement.

**Intervention**

The innovation evolved into the so called ABC Hospital Payment System, referring to a case based system which distinguished three service variants per condition. For each variant, the county designed a clinical pathway and a corresponding standardized hospital payment. Moreover, the actual services provided where closely monitored and deviations from the pathways were penalized to encourage standardization, efficiency and cost control. The initial 2011 ABC system comprised of 14 disease pathways for the THCs and 40 pathways for county hospitals. In 2013 the number of pathways had grown to 56 for THCs and 106 for county hospitals. In 2014, Xi Xian had also introduced 21 pathways for hospital outpatients, while additional pathways and adjustments where again being discussed for THCs and even for VCs.

**Results**

The overall performance results achieved in Xi Xian are summarized in the Henan Provincial Health and Family Planning Commission report (October 2014), which shows that the percentage of men indicating to be satisfied or very satisfied with the provided services increased from 41.7% to 91.5%, and similar results for women. Moreover, at 45.5 percent, the Out of Pocket payments were already low in Xi Xian in 2008 when compared to other counties in 2008. They were reduced to 12.7% by the end of HXI, much lower than all other Henan HXI counties, and the overall HXI average.

Although the ABC system has achieved considerable results from early 2012 onwards, it struggled to be successful from 2009 to 2011. It had difficulties in gaining acceptance at service delivery level, causing a low initial uptake. The difficulties appear to have been related with the resistance to give up old revenue models for new and less generous ones and with an initial lack of grass roots level involvement. It has taken sustained and collaborative efforts, and several rounds of adaptations before the innovation developed into the ABC system that was accepted among all relevant stakeholders and successfully expanded.

**Box 2:** **Case Study 2: Performance Based Management in Mei Xian, Shaanxi Province**

**County characteristics**

Mei Xian, in Shaanxi Province, located in Central China, covers 863 km^2^, and has a population of 310,000. While it is mostly flat, it has some more mountainous areas, where traveling is more difficult for patients and health workers. The 2009 per capita net income for farmers in Mei Xian amounted to 4,105 RMB.

**Baseline assessment**

In their baseline assessment of 2008 for health XI, Mei Xian, identified a number of performance problems that they had already been intending to address. A strong increase in cost of both inpatient (+28%) and outpatient visits (+11%) since 2006, while the number of visits decreased by 22%. Over prescription of medicine, in particular antibiotics, and many unnecessary diagnostic services. Moreover, the effectiveness of the (public) health service delivery remained unclear. A lack of proper incentives was seen as a major cause of these problems.

**Intervention**

To address these performance problems Mei Xian introduced a Performance Based Management (PBM) system. It created an information system which made health service delivery transparent and subsequently rewarded appropriate delivery. To this purpose it implemented aligned financial incentives for township health clinics, village health clinics (VC’s), and for personal salaries. The intervention required well defined quality indicators and a system to capture data on these indicators in a reliable and systematic way. The indicator sets were adjusted for mountainous areas. Initially, the indicator set included 168 indicators and was considered as too complex to serve as a management instrument. By the end of HXI, the indicator set consisted of 44 indicators with a total weight of 300 points.

**Results**

Comparing the years 2008 and 2013, the expenses for outpatient treatment decreased in Mei County with 38%, despite inflation, salary increases, and indeed against rising average outpatient treatment costs at the provincial level. The self-payment ratio for hospitalization decreased from 78.9%, highest among the Shaanxi HXI counties in 2008, to second lowest at 50.3% in 2013. While in 2008 the number of outpatients which was satisfied about the health services was already highest among the Shaanxi HXI counties in 2008 at 70.8%, Mei Xian remained to have a considerably higher outpatient satisfaction score of 92.4 being (very) satisfied.

**Box 3: Case study 3: Continuous improvement of public health services in Jiulongpo, Chongqing**

**County Characteristics**

Jiulongpo District is located south west of the city centre of Chongqing (population around 29 million), which is one of the four national central cities in China. It has a history as a mining district, while industry plays a main role in its current economy. The total surface of Jiulongpo is 442 km^2^, of which 362 km^2^ is considered as rural. Jiulongpo’s population is 1.141 million, and about 185,000 of them are farmers. Jiulongpu was initially not included in HXI and lobbied extensively and successfully to participate.

**Baseline**

The existing health system in 2008 hardly reimbursed preventive services. Component 3 of HXI targets preventive services, as they are considered to benefit health and reduce county level health system cost. In a condensed form, Jiulongpo is confronted with the societal problems taking place in the municipality Chongqing and the People’s Republic of China at large: the urban/rural gap, migration from rural to urban, and several increasingly vulnerable subpopulation. According to the Jiulongpo health information, the specific needs of local residents revealed by the baseline surveys are the large incidence of gastro-intestinal diseases, measles, non-communicable diseases (NCD’s) such as hypertension and diabetes, TB, HIV and mental health care.

**Intervention**

While Jiulongpo has implemented many public health service innovations, this case study rather emphasizes the underlying innovation of continuous improvement. Respondents in Jiulongpo refer to the adopted continuous improvement methods using a variety of metaphors, such as ‘master key’ and ‘roadmap’. They considered these methods as the most important project learnings. Continuous improvement, referring to process of identifying an area for improvement through (base line) measurement, analysing the situation, developing a solution, implementing it, and measure again, was a new methodology and hence an intervention it itself.

**Results**

The ratio of public health staff was 1 per 1,000 of population before the Health XI project. It was 1.4/1000 in 2009 and has grown to 1.7/1000 in 2012. The per capita funding was topped by 6 RMB for both the rural and urban population to a level of 15 RMB in 2009. It further increased over the years 2010-2014 to respectively 20, 29, 33.5 and 38 RMB; above the national target of 35RMB.The available HXI funds for investments in clinics have led to renovation and standardization of village clinics. In addition, a management information system has been implemented in the village clinics, focused on documentation and reporting. Considerable improvements on a variety of specific public health service processes and outcomes corroborate the claims on general improvement abilities obtained [9].
