# Supplementary material for: ‘Hybrid’ top down bottom up health system innovation in rural China: A qualitative analysis
Source: PLoS One. 2020 Oct 7;15(10):e0239307. doi: 10.1371/journal.pone.0239307 (PMC7540887; doi:10.1371/journal.pone.0239307)
Supplement: Attachment — Submitted filename: Review PLOS PONE-D-19-27227.pdf [file pone.0239307.s007.pdf]

Review number PONE-D-19-27227

Authors of paper: De Klundert et al.

Thank you for the opportunity to review your paper. I found resonance with my own work.

Summary of the research

The research team set out to explore the implementation of a bottom up process of reform in rural China in a context of a traditionally hierarchical system. A 'help it happen' approach was adopted, distinctive in its bottom up approach to reform implementation, as different from a traditional top down approach to implementation.

Major comments:

Introduction

Page 3, line 42 – 43

Please include what these health system reforms hope to achieve. Please add at least five words describing the overarching goal of health systems reform in China is. ….. "a series of national health system reforms *to achieve XXXXX* and achieved considerable improvements in areas such as XXXXXXX (1.2}

Other comments on introduction:

The introduction primarily speaks to literature on China, please provide a definition of 'bottom up approach' with some international empirical examples of what typically happens in a bottom up approaches to implementation, you might also want to clarify for the reader what you mean by top down implementation. This will help the Reader to situate the work in broader literature on bottom up implementation and why this work is unique. Not all readers will know what top down reform/implementation versus bottom up reform/implementation is so it would be good to situate the work – in all implementation of policies bottom up processes happen as implementers inevitably use their discretion in implementation, the difference in your study is that bottom up processes were intentional. Perhaps just one paragraph with a few key definitions and some empirical conclusions on bottom up innovations in other contexts. You could add this to paragraph two.

Please define what 'help it happen approach' is. What are the key steps or principles? In your context it seems that there are pre-set targets and there is a requirement that evidence-based innovations are to be used when counties select innovations? Also a huge expert presence in this model. This bottom up model is unique and the Reader should know this. The authors do note it is based on the principle of 'adaptation' but it would be good to know a bit more about this process specifically.

Page 4, line 85

I struggled with the fact that the research question was a 'what' question - this is typically a quantitative style of research question. This research, being qualitative in nature was more exploratory trying to identify how and why success was achieved. I suggest rephrasing the question to: *How and in what contextual conditions did a bottom up reform process achieve success in three rural Chinese districts?*

This reflects a more appropriate exploratory study which this is, also given the importance of understanding context when presenting qualitative work.

Page 4, line 90

Please make clear in the first paragraph to the Reader what the unit of analysis is in this paper. Are you primarily exploring the routinisation and adoption of a bottom up process of designing local innovations? Or are you primarily exploring the implementation of health reforms? Please link back to you research question.

Good to be explicit to the Reader what is the primary object of analysis.

The process of trying to introduce bottom up processes (in a context of typically top down hierarchy) is different from implementing reforms, even though they are linked. Good to also make link clear.

Page 4, line 97

Please explicitly include the selection criteria for the three districts. How and why were these three selected from all the best practise counties.

Page 6, line 136 - 144

The application of theory and the use of conceptual frameworks is what allows qualitative research to be transferable to other contexts, there is currently too little information on the key concepts and theory applied. Please provide more detail on why the Greenhalgh f/work was used and what specifically concepts were used in this study, please define the key concepts. Please explain why the model by the World Bank was used and why you felt it relevant to merge the two (merging is fine but the Reader should know why this specific two were merged). Please provide an explanation of what is meant by "enriched with topics from organisational culture and learning". What do these concepts mean and how is it relevant to be used in this paper. While these concepts may be intuitive to some from the social sciences, the PLOS audience goes beyond the social sciences. Also given that these concepts are used in analysis this will further support deep understanding of the relevance of the work.

In Appendix 4 a topic listing of words is insufficient, please include a definition of each of the concepts alongside the word. The Reader should be able to see a definition of each concept while they are reading to help understand the content.

Page 7, line 167

Please input a table that lists the 22 indicators, I felt as though I needed to know what this was to fully understand the intentions of the reform. Tables typically don't add to word count so this shouldn't be written in paragraph form but rather in a Table.

Page 7, 169

I am confused by the statement 'self -formulated priorities', given that they were asked to achieve a specific set of 22 indicators which means that the targets/priorities were already set by national. I think that what you are trying to say is that they were given the authority to develop bottom up interventions to achieve the pre set targets? If you could clarify that would be good.

You also state in the next paragraph 'prioritised improvement targets'.

Then on page 9, line 223 the authors write "self-set improvement targets". Is this a selection from the 22 target indicators?

Only on page 10, line 256 is the Reader made aware that the counties could add targets. I am now confused as to whether the targets/priorities spoken about earlier in the results section are referring to these new targets or the old targets.

Page 7, line 183

Could you specify if counties were expected to only develop evidence-based interventions using international best practice? Or were they also allowed to design innovations based on an understanding of their own context and to think outside of the [evidence-based] box?

This is an important point to understand the nature of bottom up innovation in this paper. One understanding of bottom up processes is where implementers craft innovations from their own understandings of context and experiences. Your paper seems to include a particular nuance on top of this.

Page 8, line 200

Spelling mistake on 'implementations'

Page 8, line 218 – 219

I am not surprised by the many iterations and adaptations, this is the nature of bottom up processes in implementation. What is interesting here is that implementers expected things to work out the first time round – this tells me that perhaps there was a lack of communication on what bottom up reform is and that in fact adaptation and learning is what should be expected.

Page 10

Suggestion: I really like that there is recognition in the paper that this model is in fact an interplay between top down and bottom up reform/implementation. It might be useful for the authors to acknowledge in the introduction that this model looks like a hybrid of top down / bottom up rather than fully bottom up. The authors do not have to do this, but it might be good to qualify this upfront. I think this links to me earlier point in the introduction section where the work needs to be situated briefly in theory on what top down implementation is versus bottom up and the inevitability of both being present.

Page 12. Line 326

I thought there were 22 indicators, and yet when I arrive at this point in the paper the Reader is advised there are 166 indicators. I am therefore confused by the lack of clarity on the number of targets etc. Please clarify and report so that the Reader is clear on which targets, set by whom, how many and in which cycle of the process in the results section.

Page 13, line 366

Please explain in which ways you understand the three counties to have been 'ready for change'. Readiness for change is a critical part of reform adoption and implementation, thus the Reader would benefit from this information. Please link this to your understanding to readiness for change as defined by Greenhalgh.

Page 13, line 365 – 374

A suggestion: I assume also based on your results that the reason it was hard in the beginning is because identifying innovations based on international literature is a very hard thing to do, local level

actors are often not trained to read empirical literature and then to rank interventions using evidence. I think if you have any knowledge about the process of the experts engaging with the international and sharing with the local actors this would be good to know to. I think it may be unfair to say only 6 out of 40 achieved this on schedule as it is a highly complex process of sorting through technical data. As mentioned, in the introduction it would be good to know what the model of bottom up implementation is – does it include evidence based innovations only or home grown also?

Page 13, line 380

Please list three examples of targets achieved. The reason I ask this is a simliar reason I asked for the table earlier of the 22 indicators, while success is mentioned in the introduction I as the Reader do not have a sense of what success was achieved, I realise that the success/benefits are published in another paper but it would be nice to have some sense within the paper. I realise more information is given in the Appendix. I am not asking for a major change in the paper in any way, just a sense here and there. The goals of the reform are listed once only in the introduction but connections are not made again explicitly to these goals in the paper, linking your findings in some way to these goals more explicitly will help the Reader stay connected to the overall goals.

The conclusion

Things to consider: please see my comment above linked to page 13, line 365 – 374 and take into consideration if my comment is relevant. I think important to note in the conclusion that while this reform process was labelled as ‘bottom up” it was in fact an interplay between top down and bottom up that drove success, which given the nature of expert support in this context is plausible.
