# Supplementary material for: ‘Hybrid’ top down bottom up health system innovation in rural China: A qualitative analysis
Source: PLoS One. 2020 Oct 7;15(10):e0239307. doi: 10.1371/journal.pone.0239307 (PMC7540887; doi:10.1371/journal.pone.0239307)
Supplement: Attachment — Submitted filename: Rebuttal final.docx [file pone.0239307.s008.docx]

Response to Review Comments / Rebuttal

We thank the editor and the reviewers for their very thorough reading of our manuscript and the many thoughtful and valuable revision suggestions. In the ‘Rebuttal Letter’ we address all the points one by one, grouping the comments of the editors and both reviewers thematically (where some comments occur in multiple themes.) We hope this approach assists the editor and the reviewers to see the interrelationships between the comments made, and to understand how the revision provides a systematic, unified response to review comments, regardless of their differences in perspectives. Together the comments have helped us to improve the positioning of the manuscript. The revised version has a complete revised introduction and methods section, and accordingly updated results, discussion and conclusions.

To help the reviewers verify that all review comments have been systematically addressed we have provided a numbering to the comments (see Rebuttal Appendix 1 attached to this letter) and refer to this numbering in the thematic grouping and ‘rebuttal’ below. The word rebuttal is in parenthesis as we have sought to address all reviewer comments, following their suggestions and addressing concerns constructively, rather than to oppose their views.

We thank you again for providing us the opportunity to revise the manuscript and hope you will find it much improved.

## Comments on Research Question and Theoretical Embedding

E0: The reviewers have provided thorough and detailed comments in an effort to strengthen the manuscript and study reporting. Please pay particular attention to the qualitative methods reporting and the integration of the theoretical framework throughout the paper. I look forward to receiving your revised manuscript.

*Thank you for providing this general guidance. Please find detailed responses on the comments regarding the theoretical framework, and more generally the positioning and embedding of the research in the introduction first, and responses to comments on the qualitative research methods in the next thematic section.*

*To address the above comment and the comments below regarding the research question, we have by and large rewritten the introduction. Firstly, it now provides extra information in the text and appendices on China’s rural health reform and HXI, to better understand the ‘approach’ taken by HXI. Moreover, the revised introduction doesn’t use the ‘help it happen’ approach as a central concept. Instead, it relies distinguishes top-down and bottom-up approaches to healthcare reform, following the initial wording of the key stakeholders of HXI and the (literature) suggestions of the second reviewer. Accordingly, and as suggested by the second reviewer, the manuscript now also uses the concept of a ‘hybrid approach’. We establish that the evidence on hybrid approaches is scarce, and validity of this evidence in the context of health reform in rural China may be limited. Hence, a revised research objective and question emerge, which are provided in the detailed answers below.*

*Addressing the comments on the research question and literature embedding coherently, and in alignment with the comments on the methods required a major revision of the introduction rather than inserting/modifying sentences or paragraphs per comment. Especially so as the two reviewers took different perspectives, and the resulting comments were non-trivial to join.*

R2.1 Page 3, line 42 – 43 Please include what these health system reforms hope to achieve. Please add at least five words describing the overarching goal of health systems reform in China is. ….. “a series of national health system reforms *to achieve XXXXX* and achieved considerable improvements in areas such as XXXXXXX (1.2}

*We have now cited a Lancet publication authored by China’s Minister of Heath in 2009, Chen Zhu, which describes the 5 pillars of the important health reform round of 2009, which is of specific relevance for our study:*

*“Within this series, 2009 formed a watershed year in which a comprehensive round of reforms consisting of five pillars was announced with the aims of ‘1) expanding the coverage of and benefits provided by subsidised health insurance; 2) implementing a drug reform scheme with zero mark-up on listed essential medicines; 3) improving access to and the quality of primary health care; 4) implementing basic public health screening and management at the community level for all; and 5) implementing public hospital reform’ [3].”*

R2.6 Page 4, line 90 Please make clear in the first paragraph to the Reader what the unit of analysis is in this paper. Are you primarily exploring the routinisation and adoption of a bottom up process of designing local innovations? Or are you primarily exploring the implementation of health reforms? Please link back to you research question. The process of trying to introduce bottom up processes (in a context of typically top down hierarchy) is different from implementing reforms, even though they are linked. Good to also make link clear.

*The unit of analysis is now explicitly specified at the end of the second paragraph:*

*“In this study, we analyse HXI’s hybrid bottom-up/top-down approach to the implementation of health reforms.”*

*As suggested by the reviewer, this is also reflected in the revised research question, which now reads:*

*“How has the effective implementation of health reform interventions occurred in the hybrid bottom-up/top-down approach taken by HXI in the context of rural China?”*

*(in which the county level perspective is implied by the design of HXI.)*

R 2.3 Not all readers will know what top down reform/implementation versus bottom up reform/implementation is so it would be good to situate the work – in all implementation of policies bottom up processes happen as implementers inevitably use their discretion in implementation, the difference in your study is that bottom up processes were intentional. Perhaps just one paragraph with a few key definitions and some empirical conclusions on bottom up innovations in other contexts. You could add this to paragraph two.

*The introduction is now rewritten in terms of top-down and bottom-up approaches and the research interest has been recast in terms of ‘hybrid’ approaches. The introduction uses definitions for all three of them, based on the literature suggested by the reviewer [9]. We believe that the notion of street level bureaucrats is less relevant for HXI, as the intervention design was intentionally bottom up (as also noted by the reviewer). We do however, elaborate on the particular approach to bottom-up health reform taken in HXI: selection and adaptation of international best practices. The corresponding adjustments are made in various paragraphs. For instance, the definition of top-down is provided in the first paragraph:*

*“‘a policy-centred and rational process that begins with an authoritative decision by senior policy officials’ [9]”*

*Bottom-up is subsequently addressed in the second paragraph which now reads:*

*“Despite the accomplishments of China’s health reform by top-down approaches, the World Bank, the British Department of Foreign Investment (DFID), and the Chinese government (i.e., The National Health & Family Planning Commission (NHFPC), the National Development & Reform Commission, and the Ministry of Finance) agreed in 2008 that the health services for the rural population were lagging behind and that their wide variety of contexts, populations, and needs had not always been served equitably by the implemented top-down reforms [4]. Therefore, these stakeholders jointly set up China’s Rural Health Reform Project Health XI [4]. The design and intention of HXI was to pilot the implementation of health system reform interventions in 40 rural counties from eight rural provinces following a ‘bottom-up’ approach [4,12-14]. This approach required each county to implement reform interventions tailored to the specific needs and context of that county. HXI took a very specific bottom-up approach in which counties were required to follow a pre-structured process of selection and adaptation of international best practices [4,12-14]. This somewhat contrasted with the more common definition of bottom-up reform, which refers to developing policy and interventions in ‘a joint process of formulation and implementation involving networks and coalitions’ [9]. In this study, we analyse HXI’s hybrid bottom-up/top-down approach to the implementation of health reforms.”*

R2.2 Other comments on introduction: The introduction primarily speaks to literature on China, please provide a definition of ‘bottom up approach’ with some international empirical examples of what typically happens in a bottom up approaches to implementation, you might also want to clarify for the reader what you mean by top down implementation. This will help the Reader to situate the work in broader literature on bottom up implementation and why this work is unique.

*The revised text gives a definition for bottom up health reform taken from the literature suggested by the reviewer [9] and makes clear how HXI was based on a specific form of bottom up reform [4,12-14]. We believe these suggestions by the reviewer have indeed improved the manuscript significantly. At the same time, we have left the discussion relating it to the existing evidence base on bottom up versus top down approaches and perspectives on healthcare reform and innovation to the Discussion section. Such to avoid introducing two different theoretical perspectives in the introduction, from which the ‘let it happen, help it happen, make it happen’ [10,11] continuum has now disappeared (yet is saved for later). The introduction can therefore better bring out the specific bottom up approach taken by HXI with the aforementioned general definition. That being said, reference to the authorative literature review of Greenhalgh et al. [10,11] and the concepts therein are already provided on several occasions in the introduction, for instance when mentioning adaptation as an element of a bottom up approach.*

R2.17 Page 10 Suggestion: I really like that there is recognition in the paper that this model is in fact an interplay between top down and bottom up reform/implementation. It might be useful for the authors to acknowledge in the introduction that this model looks like a hybrid of top down / bottom up rather than fully bottom up. The authors do not have to do this, but it might be good to qualify this upfront. I think this links to me earlier point in the introduction section where the work needs to be situated briefly in theory on what top down implementation is versus bottom up and the inevitability of both being present.

*Thank you for your constructive comment. The introduction now follows the theoretical embedding proposed by the reviewer. The introduction now explicitly mentions that HXI uses a blend of bottom up and top down approaches in the 3^rd^ paragraph where we also implicitly define ‘hybrid’ approaches:*

*“HXI’s bottom-up process was guided by extensive formal planning and control structure and contractually agreed to by The World Bank, DFID, and China’s national government, thus complementing ‘bottom-up’ elements with ‘top-down’ elements.* *In this study, we analyse this ‘hybrid’ process of top-down and bottom-up implementation of health reforms in rural China.”*

R2.4 Please define what ‘help it happen approach’ is. What are the key steps or principles? In your context it seems that there are pre-set targets and there is a requirement that evidence-based innovations are to be used when counties select innovations? Also a huge expert presence in this model. This bottom up model is unique and the Reader should know this. The authors do note it is based on the principle of ‘adaptation’ but it would be good to know a bit more about this process specifically.

*The introduction of ‘help it happen’ is now postponed to the Discussion section. This helps to overcome the difficulty that there is no explicit definition for a ‘help it happen’ approach’ from the literature, and we therefore describe elements of it rather than defining it.*

*Between ‘let it happen’ and ‘make it happen’ Greenhalgh et al. [10,11] position the ‘help it happen’ perspective which emphasizes negotiation, influencing and enabling.*

R1.2. Research question: even though current literature showed the effectiveness of Health XI project somehow in China, it does not mean "bottom-up" reform happened in China. There might be many other factors/interventions contributing to the positive result of the Health XI project.

*We thank the reviewer for his critical view on the validity of the evidence regarding the effectiveness of HXI and on the attribution to the bottom up nature of the approach in particular. These are important and valid considerations. The revised manuscript addresses both these concerns more explicitly. The introduction now more clearly states that the evidence identifies that HXI counties have performed better in comparison to a set of control counties, as recently published after a difference in difference analysis [15]:*

*“A quantitative difference in difference study provides evidence that, in comparison to carefully selected rural control counties, HXI counties have achieved ‘substantial benefits given the relatively limited investment’ [15]. These benefits include ‘significant decreases in outpatient expenditure and financial strain, and improvements in public health services provision, as well as in two out of five dimensions of the EQ5D model for health-related quality of life (mobility, and pain/discomfort)’ [15]. Evidence of these results is further strengthened by evaluation studies of specific interventions [14,16,17]. The official evaluation by the World Bank reports that, with one exception, app performance targets on the 22 project indicators were either ‘achieved’ or ‘surpassed’ [4].”*

*We also hope that the revised introduction which more clearly describes the mandatory bottom up features of HXI, further laid out in references [4,12-14], the case reports provided in [20] (see also Appendix 5), and Table 1 of [15] convincingly witness that bottom up reform has happened within HXI.*

*This leaves open if and how the bottom-up elements of HXI have contributed to the effectiveness of HXI. Indeed, other aspects of HXI may have determined the effectiveness. The revised research question doesn’t assume the bottom-up elements are responsible for the effectiveness, but rather asks*

*“How has the effective implementation of health reform interventions occurred in the hybrid bottom-up/top-down approach taken by HXI in the context of rural China?”*

*Please note that this explorative research question now mentions a hybrid approach, rather than a bottom-up approach, following the comments of Reviewer 2. The formulation of this explorative research question will be readdressed below when considering your valuable comments on the research methods, which have been completely rewritten as well.*

R2.5 Page 4, line 85 I struggled with the fact that the research question was a ‘what’ question - this is typically a quantitative style of research question. This research, being qualitative in nature was more exploratory trying to identify how and why success was achieved. I suggest rephrasing the question to: *How and in what contextual conditions did a bottom up reform process achieve success in three rural Chinese districts?* This reflects a more appropriate exploratory study which this is, also given the importance of understanding context when presenting qualitative work.

*Following the suggestions of the reviewer, and to better address the concerns of Reviewer 1 (see preceding comment), we have reformulated the research question as a ‘how’ question along the lines you have suggested. Following your earlier suggestion to use the theoretical notion of hybrid approaches, and to avoid presupposing that the bottom up elements have been instrumental to ‘achieve success’ (see previous comment by Reviewer 1), the reformulation has become:*

*“How has the effective implementation of health reform interventions occurred in the hybrid bottom-up/top-down approach taken by HXI in the context of rural China?”*

*Hopefully the editor and both reviewers are willing to accept the revised research question which explicitly and jointly takes all their comments into account, and now emerges from the HXI design notions and a simplified theoretical perspective of top down, bottom up, and hybrid approaches. The explorative nature of this research question and simplified theoretical notion have been of explicit and significance importance when (completely) revising the Research Methods section, whose revision is addressed next.*

## Comments on Research Methods

E0: The reviewers have provided thorough and detailed comments in an effort to strengthen the manuscript and study reporting. Please pay particular attention to the qualitative methods reporting and the integration of the theoretical framework throughout the paper. I look forward to receiving your revised manuscript.

*In response to this comment, which also started the review response in the previous session, let us first recall here that the research question has been revised following the suggestions of Reviewer 2. The resulting ‘how’ question is an explorative question, addressing how the hybrid approach taken by HXI has functioned in order to produce the effects in the rural Chinese context.*

*This explorative question does not seek to validate consisting conceptual models. As pointed out by Greenhalgh [11], the validity of existing models for health service innovation - including hybrid approaches - is limited, and the introduction argues why this is even more the case for rural China. Hence the explorative nature of the revised research question.*

*By consequence, our qualitative research methods don’t follow a deductive approach (as suggested by Reviewer 1) in which a conceptual model derived from literature (theory) is presented and the data analyses seeks to verify or falsify the conceptual model, possible leading to adjustments and extensions. Neither is our approach entirely without theory, and therefore a purely inductive approach in which the theory emerges from the data [26] would be ill suited as well. As now explicitly and extensively argued in the revised methods section, the revised research question calls for an approach which combines inductive and deductive steps which uses supporting concepts in deductive stages. This approach is far from a novelty, As Thomas [26] writes: “in practice, many evaluation projects use both inductive and deductive analysis.”*

*The thoughtful feedback of both reviewers has helped us sharpen and clarify the methods used and communicate them accordingly in the revised manuscript, with supporting references [24,25,26]. We are thankful to both reviewers for their constructive criticism.*

*As a result of this repositioning, the evidence based model of health services innovation which remains to be presented in Figure 1 doesn’t function as the conceptual model. Instead it functions to support the interviews, ensuring that all stages of diffusion/dissemination of innovation and their relevant components [10,11] are covered in the interviews.*

*We apologize for this long initial statement, which we consider necessary to motivate the revisions made as required to hopefully jointly address the concerns of both reviewers, in a methodologically sound manner.*

R1.8 The authors used a conceptual model of innovation in the analysis. But as a qualitative research, the original model should be deduced, deepened and finalized into a new model.

*We thank the reviewer for this important comment as it made clear to us that the model in Figure 1 didn’t actually play the role of a conceptual model and shouldn’t have been presented as such. In the revised version it is not, and in fact in the revised version there is no explicit conceptual model to start from. We hope that the comments above and the revised initial paragraph of the methods section convince the reviewer that this befits the explorative research question:*

*“In alignment with the research question, this study is of an explorative nature and follows a matching sequence of inductive and deductive steps [24]. Based on an initial exploration of our cases (inductive), we identified relevant (guiding) concepts from the literature to further sharpen the research aims, research question and methods, and specifically the interview questions (deductive). During the analyses, the data were first categorized based on the guiding concepts (deductive). Subsequently, an inductive approach was used to analyse the data and identify relevant themes [24-26].”*

*As is hopefully clear in the revised version, the presented research is not deductive and therefore shouldn’t necessarily ‘deduce, deepen and finalize’ an original conceptual model ‘into a new model’. Still, the explorative question and combined inductive and deductive approach can add to existing models. In addition, it may propose new models and concepts to explain how the hybrid approach of HXI has worked out. The revised methods section now elaborates the methods and their justification much more extensively and in better alignment with the research question.*

*We might notice already that the Discussion section explicitly reflects on the contributions of the explorative findings to existing models and evidence on hybrid approaches. One might indeed take the viewpoint that the findings ‘deepen’ the understanding of hybrid approaches and how they may use the continuum from bottom up to top down approaches, distinguishing a procedural dimension from a content dimension.*

*We hope the revised introduction and methods sections address the concerns of the reviewer, and that the reviewer finds the corresponding adjustments made to the results and discussion to be strengthening the manuscript.*

R2.8 Page 6, line 136 – 144. The application of theory and the use of conceptual frameworks is what allows qualitative research to be transferable to other contexts, there is currently too little information on the key concepts and theory applied. Please provide more detail on why the Greenhalgh f/work was used and what specifically concepts were used in this study, please define the key concepts.

*Let it be noted first that in the revised version, the framework of Greenhalgh (Figure 1) doesn’t serve anymore as the central conceptual model. Nevertheless, its relevance is introduced and more extensively motivated in the methods section:*

*“The initial analysis further revealed that the World Bank provided and taught a model of health reform stages to support counties in their implementation processes [4]. This staged model closely resembled the well-known evidence-based framework for the spread of health service innovations in Greenhalgh [10,11]. By merging the terms of the World Bank model into the evidence-based framework, the model depicted in Figure 1 arose. This merged model, which emphasizes the staged and cyclic nature of health system reform, supported the interviewing process, as it enabled us to collect data following an evidence-based framework that completely captures the stages of implementation of innovations using terminology that HXI participants were already familiarized with. The model contains a subcycle formed by the stages of adaptation, implementation, and evaluation, recognizing that various cyclic iterations might be conducted per intervention.”*

*The key concepts of hierarchy and power distance (relating to bottom up approaches in a top-down culture) and learning paradigms and practices (knowledge transfer versus knowledge discovery) remain positioned towards the end of the introduction. Moreover, in the revised methods section, it now becomes clear how they resulted from the initial explorative data collection activities. The concepts of top down, bottom up and hybrid approaches are now explicitly defined in the introduction.*

R2.9 Please explain why the model by the World Bank was used and why you felt it relevant to merge the two (merging is fine but the Reader should know why this specific two were merged). Please provide an explanation of what is meant by “enriched with topics from organisational culture and learning”. What do these concepts mean and how is it relevant to be used in this paper. While these concepts may be intuitive to some from the social sciences, the PLOS audience goes beyond the social sciences. Also given that these concepts are used in analysis this will further support deep understanding of the relevance of the work.

*The answer to the first subquestion (*why the model by the World Bank was used and why you felt it relevant to merge the two (merging is fine but the Reader should know why this specific two were merged) *is now explicitly provided in the lines 136-144, as also addressed in the response to the previous comment.*

*The phrase “enriched with topics from organisational culture and learning” has been deleted from the paper. The corresponding concept remain motivated and elaborated in the introduction and the methods section as explained in the answer to the previous comment.*

R2.7 Page 4, line 97 Please explicitly include the selection criteria for the three districts. How and why were these three selected from all the best practise counties.

*The selection process is now explicitly described as follows:*

*“The three counties were selected by the researchers from three lists of counties that were considered best-practice counties by the HXI project management office CPSM. Each of these three lists included best-practice counties for one of the three reform components of HXI. Xi Xian was selected for its innovative hospital financing reform, Mei Xian was selected for its comprehensive service delivery management reform, and Jiulongpo was selected for its achievements in public health improvement. Moreover, the three counties together covered the included provinces relatively well from a geographical and economic perspective. The method of selecting only from the supposedly effective counties does not allow a full evaluation of HXI; it does, however, suit the research objective of exploring how the hybrid approach of HXI worked in those counties where it has been effective.”*

R1.3 Sampling strategy: the criteria for selection of research participants is not clear. Meanwhile, the authors did not say how to achieve information saturation. It seems the sampling is random. A minor question: in the text, it says 3 group interviews were conducted (Line 105). But in Table 1, there shows 4 focus group discussions.

*We regret that the sampling strategy was not clear to the reviewer in the original manuscript. We hope, the revised manuscript makes this much clearer. As stated above, the sampling started with selecting counties:*

*“The three counties were selected by the researchers from three lists of counties that were considered best-practice counties by the HXI project management office CPSM. Each of these three lists included best-practice counties for one of the three reform components of HXI. Xi Xian was selected for its innovative hospital financing reform, Mei Xian was selected for its comprehensive service delivery management reform, and Jiulongpo was selected for its achievements in public health improvement. Moreover, the three counties together covered the included provinces relatively well from a geographical and economic perspective. The method of selecting only from the supposedly effective counties does not allow a full evaluation of HXI; it does, however, suit the research objective of exploring how the hybrid approach of HXI worked in those counties where it has been effective.”*

*Next, we sampled respondents as follows:*

*“Respondents were selected from the international parties involved in the health reforms and from multiple levels within China, i.e., the national level, the provincial level, the county level, the organizational level, and the professional level. Moreover, the researchers conducted group interviews with patients. In addition, international-, national-, provincial-, and county-level experts who were involved were selected for interviews. Respondents from the World Bank, DFID, and the National Health and Family Planning Commission were selected and contacted directly by the researchers for interviews.* *The CPSM, in collaboration with provincial and county health bureau administrators, selected and invited for interviews the respondents indicated by the researchers at the local level. Likewise, the CPSM selected experts for interviews and provided logistic support. Table 1 gives a detailed account of all the respondents.”*

*The sampling is therefore purposive, covering all levels of HXI (including patients) to completely elicit the working of top down and bottom up approaches, and covering each of the three reform areas of HXI. The sampling has been far from random, as has been made clear in the revised manuscript.*

*The topic of saturation is now also extensively and explicitly addressed in the revised manuscript, in line with the present methodological discourse on this topic [27]:*

*“Given the size of HXI, which encompassed 40 counties in eight provinces in which more than 200 innovations have been implemented and which covered a population of more than 20 million citizens [15], completeness of data collection or analysis, for instance, as expressed through various forms of data saturation criteria [27], is hard to obtain. This problem persists when restricting the research design to three counties. In combination with the practical delays between data collection, transcription, translation and analysis, a classical criterion of data saturation was therefore difficult to adopt. Instead, we relied on alternatives that matched our research approach, which combined inductive and deductive steps. From the broader perspective of completeness, let us first note that we sampled counties from each of the three reform areas of HXI and respondents from all levels of HXI, including patients. As much as possible (e.g., due to obtaining consent from respondents), we interviewed multiple respondents of each relevant level and type. For some levels, only one respondent existed. Our interview protocol and coding based on guiding concepts ensured a priori thematic saturation of the deductive analysis steps [29]. The open coding and joint iterative analyses by DdK and JvW in the inductive stages converged towards inductive thematic saturation [29].”*

R1.4 Interviewers: because qualitative researchers themselves are research tools, it is important to state in detail who did the interviews: do they have any experience in qualitative study? Have they both been trained for this study? How did the two interviewers speaking different language work together? Also, who did data analysis? How did the researchers do data analysis?

*As is now stated explicitly in the revised version under “data collection”, the interviews were done by Yuan Shasha, Wang Fang, Dirk de Korne and Joris van de Klundert, the first four authors of this manuscript. Hence there were four interviewers. As is now more clearly explained the interview questions were always stated in English:*

*“The face-to-face interviews with the Chinese respondents were always conducted by a pair of researchers, one of whom was of Chinese nationality (FW or SY) and one of whom was from abroad (JvdK or DdK). These interviews were conducted as follows:*

1. *The researchers’ questions were always asked in English;*
2. *A certified interpreter translated the questions into Chinese and then asked them to the respondents;*
3. *The respondents answered in Chinese;*
4. *The certified interpreter then translated the responses from Chinese to English; and*
5. *The next question was then asked, etc.*

*On two occasions, the interview was moderated by SY instead of a certified interpreter.”*

*All interviewers have PhDs and held academic positions at highly ranked universities in the domain of health sciences, as can be deduced from the author information. Moreover, they have experience with qualitative research methods, as evidenced by Rebuttal Appendix 2 which contains a list of publications presented qualitative research by the authors/interviewers in peer reviewed international scientific journals. Many of the publications have appeared in highly ranked health policy and management journals such as the BMJ Open, Human Resources for Health, Journal of Medical Internet Research, BMJ Global Health, BMC Health Services Research, et cetera. The list includes several researchers in China, and a publication in a Chinese journal.*

R2.10 In Appendix 4 a topic listing of words is insufficient, please include a definition of each of the concepts alongside the word. The Reader should be able to see a definition of each concept while they are reading to help understand the content.

*The extensive topic list with sample questions (including Chinese translation of concepts presented to support the interpreters) is now provided in Appendix 5. It gives much more detail on how the specifics of the topics (which are defined in Greenhalg et al. [11]) can be operationalized. It should be noted that depending on the respondent, only a subset of the topics and proposed questions typically appeared relevant, which were then further elaborated via open questions and probing.*

R1.5 Working language: it is very confusing. Regarding interviews, “these interviews were conducted in Chinese using certified interpreters who interpreted consecutively” (line 122-123). Does it mean the Chinese interviewer did the interviews in Chinese, and a translator did translation during the interview? Or how did you organize the interviews? It is not clear in the manuscript. Regarding data analysis, “the English texts of the interviews were transcribed using audio files of the original and translated interview content” (line 150-151). If the interviews are bilingual, how were they been transcribed and translated? This should be described more clearly in the paper.

*Instead of simply mentioning consecutive translation, the revised manuscript now provides explicit detail on the interview translation process. We thank the reviewer for this suggestion!*

*“The face-to-face interviews with the Chinese respondents were always conducted by a pair of researchers, one of whom was of Chinese nationality (FW or SY) and one of whom was from abroad (JvdK or DdK). These interviews were conducted as follows:*

1. *The researchers’ questions were always asked in English;*
2. *A certified interpreter translated the questions into Chinese and then asked them to the respondents;*
3. *The respondents answered in Chinese;*
4. *The certified interpreter then translated the responses from Chinese to English; and*
5. *The next question was then asked, etc.”*

*Regarding the transcription, the revised manuscript now more clearly states that:*

*“The English texts of the interviews were transcribed using audio files of the full interviews. The Chinese audio content was used to resolve unclarities in the English text when transcribing.”*

*Thus, as is now hopefully fully clarified in the manuscript, the translation took place during the interview, of which the translated English parts were transcribed. We thank Reviewer 1 for pointing that these processes required clarification.*

R1.6 Ethics: because the study involved human participants, please list the name of the IRB or ethics committee and other information of the ethical approval. Otherwise, you should give sufficient information for why not obtaining ethical approval.

*See the response to the editor on the corresponding comment on consent to below.*

R1.7 The trustworthiness of the results has been affected by the vagueness of the research method.

*We hope that together with the modification of the theoretical embedding, the research question, the extensive explanation of the combined inductive -deductive approach, and the revisions on the methods section as a whole, the reviewer now agrees that the research methods are clear and appropriate to produce trustworthy results.*

E2. When reporting the results of qualitative research, we suggest consulting the COREQ guidelines: <http://intqhc.oxfordjournals.org/content/19/6/349>. In this case, please consider including more information on the number of interviewers, their training and characteristics; how participants were selected; if a pilot study was tested; how data was coded; if bias issues were considered.

*We thank the editor for the suggestion to use the COREQ guidelines [29] from 2007. As indicated in the original manuscript we have instead used the 2014 SRQR (Standards for Reporting Qualitative Research) [28] which are based on COREQ as well as 39 other sources for reporting qualitative research. Hence, we believe SRQR is preferable and hope that the editor agrees with out choice to present these guidelines. More so, as these have actually been used when advancing with our analyses and researching. The second paragraph of the methods section has been adjusted to clearly communicate our choice to use SRQR:*

*“The methods and reporting follow the SRQR protocol for reporting qualitative research (which encompasses the COREQ guidelines [27, 28]). The full SRQR checklist is in Appendix S3. Data were collected using document analyses, observations, individual interviews and group interviews. “*

*As is now explicitly clarified in the revised manuscript, the interviews were conducted in pairs by four of the authors, all of whom are experienced and widely published qualitative health services researchers with PhD’s (see Rebuttal Appendix.) Participant selection is now also more clearly explained:*

*“Respondents were selected from the international parties involved in the health reforms and from multiple levels within China, i.e., the national level, the provincial level, the county level, the organizational level, and the professional level. Moreover, the researchers conducted group interviews with patients. In addition, international-, national-, provincial-, and county-level experts who were involved were selected for interviews. Respondents from the World Bank, DFID, and the National Health and Family Planning Commission were selected and contacted directly by the researchers for interviews. The CPSM, in collaboration with provincial and county health bureau administrators, selected and invited for interviews the respondents indicated by the researchers at the local level. Likewise, the CPSM selected experts for interviews and provided logistic support. Table 1 gives a detailed account of all the respondents.”*

*In addition, the directly preceding paragraph in the methods section now more clearly explains the selection of best practices counties.*

*As is now explained in the first paragraph of the completely revised methods section, a pilot study has been done, and we used sequence of inductive and deductive analysis steps (which are later described to imply a combination of thematic and open coding):*

*“In alignment with the research question, this study is of an explorative nature and follows a matching sequence of inductive and deductive steps [24]. Based on an initial exploration of our cases (inductive), we identified relevant (guiding) concepts from the literature to further sharpen the research aims, research question and methods, and specifically the interview questions (deductive). During the analyses, the data were first categorized based on the guiding concepts (deductive). Subsequently, an inductive approach was used to analyse the data and identify relevant themes [24-26].”*

*“The interview and document data were sequentially coded and categorized using labels from our guiding concepts (deductive) and open labels (inductive) by DdK and JvW. The labels were used to identify recurrent themes.”*

*A conscious decision has been made to collect data in ‘best practice’ counties, one for each of the three reform areas, to gain understanding of how the hybrid approach has been effective. Obviously, this brings about some limitations as well as is now reworded in the opening sentences of the paragraph addressing limitations:*

*“While our research design has provided an in-depth analysis in the three selected counties and at HXI project level, it also has limitations. We have not been able to collect data in counties not participating in HXI, or in HXI counties that are reported to have had difficulties with realizing the objectives [9,17].”*

E5. Please provide additional details regarding participant consent. In the ethics statement in the Methods and online submission information, please ensure that you have specified (1) whether consent was informed and (2) what type you obtained (for instance, written or verbal). If your study included minors, state whether you obtained consent from parents or guardians. If the need for consent was waived by the ethics committee, please include this information.

*As stated explicitly in the manuscript all respondents gave explicit informed consent verbally on audio tape, based on anonymity:*

*“All respondents provided their explicit consent to be interviewed verbally and on record, as well as for interviews to be recorded for the purpose of anonymous data collection. Such after having accepted the invitation to participate and being made aware that the data collection was part of the external evaluation of HXI commissioned by the World Bank.”*

*It is because of this anonymity and the small set of candidates the respondents were selected from that we cannot provide further data on age, gender, et cetera.*

E9. Your ethics statement must appear in the Methods section of your manuscript. If your ethics statement is written in any section besides the Methods, please move it to the Methods section and delete it from any other section. Please also ensure that your ethics statement is included in your manuscript, as the ethics section of your online submission will not be published alongside your manuscript.

*The Ethics statement in the methods section has been revised according to the suggestions above and now reads (repeating some of the content from the response to the previous comment:*

*“Each interview lasted between one and three hours. All the respondents provided their explicit consent to be interviewed verbally and on record, as well as for their interviews to be recorded for the purpose of anonymous data collection, after having accepted the invitation to participate and being made aware that the data collection was part of the external evaluation of HXI commissioned by the World Bank. The interviews never discussed patient cases or individual health-related information. Both project HXI itself and the evaluation study were approved by the World Bank and the National Health & Family Planning Commission.”*

## Comments on HXI Clarification

R1.1. Background: please give more details of the intervention package of Health XI project in order to help the audience to better understand what the role and importance of "help it happen" approach in the project.

*We appreciate this comment and believe giving examples of the interventions early on helps to reader to develop an understanding of HXI. Hence, we have made the following addition to the (now 4^th^ paragraph) of the introduction:*

*“Examples of selected and adapted interventions for each of the three areas are 1) improving NCMS coverage and integration with MA (to improve rural health financing), 2) introducing new hospital payment systems (e.g., case-based payment) and introducing performance-based payment in hospitals and lower-level facilities, and 3) training public health workers as well as the population on hypertension and diabetes management. See Table 1 of [15] for a comprehensive overview.”*

*This addition also provides a reference to a more extensive intervention synthesis available in open access scientific literature. As is now hopefully clearer from the revised introduction, HXI didn’t prescribe an ‘intervention package’.*

R2.11Page 7, line 167 Please input a table that lists the 22 indicators, I felt as though I needed to know what this was to fully understand the intentions of the reform. Tables typically don’t add to word count so this shouldn’t be written in paragraph form but rather in a Table.

*This list is now provided as S1 Appendix 1: List of HXI Indicators.*

R2.12 Page 7, 169 I am confused by the statement ‘self -formulated priorities’, given that they were asked to achieve a specific set of 22 indicators which means that the targets/priorities were already set by national. I think that what you are trying to say is that they were given the authority to develop bottom up interventions to achieve the pre set targets? If you could clarify that would be good. You also state in the next paragraph ‘prioritised improvement targets’.

Then on page 9, line 223 the authors write “self-set improvement targets”. Is this a selection from the 22 target indicators?

*The improvement targets are self-formulated in several ways. First of all, for each of the indicators, the counties could set targets for the targeted performance level themselves (e.g. for the indicator ‘% of deliveries by caesarean section’, one county can set the target of reducing it to 30%, while another can target to keep it at 50% max). Second, one county can choose to work on this indicator from the first year, where another county can leave it for later. Thirdly, counties can propose targets which are more indirectly related to the 22 indicators, e.g. regarding the number of implemented clinical pathways.*

*We have made changes to the corresponding text in the Results section. To start from line 266:*

*“The counties were subsequently expected to select, adapt, and implement health system innovations (sometimes referred to as interventions below) according to yearly plans developed at the county level to address the priorities they formulated themselves as being necessary to meet their specific health needs. These priorities needed to cover the 22 HXI indicators and might have addressed additional performance measures as well. Each county was subsequently required to report measurable progress in each of the three HXI areas: 1) health system financing, 2) service delivery, and 3) public health.”*

*The sentence referring to the ‘prioritized improvement targets’ is rewritten to reflect that the workshop presented interventions to improve on the 22 HXI indicators (line 273 onwards):*

*“Moreover, HXI provided the counties with an initial series of workshops in which international experts transferred knowledge about (evidence-based) best practices to improve performance on the HXI indicators. For each county, funding was conditional on their initiating and realizing planned achievements.”*

*We hope this clarifies the concerns raised by Reviewer 2.*

R2.13 Only on page 10, line 256 is the Reader made aware that the counties could add targets. I am now confused as to whether the targets/priorities spoken about earlier in the results section are referring to these new targets or the old targets.

*This is now resolved in the revised text of line 266 onwards:*

*“The counties were subsequently expected to select, adapt, and implement health system innovations (sometimes referred to as interventions below) according to yearly plans developed at the county level to address the priorities they formulated themselves as being necessary to meet their specific health needs. These priorities needed to cover the 22 HXI indicators and might have addressed additional performance measures as well. Each county was subsequently required to report measurable progress in each of the three HXI areas: 1) health system financing, 2) service delivery, and 3) public health.”*

R2.14 Page 7, line 183 Could you specify if counties were expected to only develop evidence-based interventions using international best practice? Or were they also allowed to design innovations based on an understanding of their own context and to think outside of the [evidence-based] box? This is an important point to understand the nature of bottom up innovation in this paper. One understanding of bottom up processes is where implementers craft innovations from their own understandings of context and experiences. Your paper seems to include a particular nuance on top of this.

*The revised introduction now clearly specifies early on that counties were expected to innovate bottom up by selecting international best practices and adapting them to their local needs and context (lines 59-64):*

*“This approach required each county to implement reform interventions tailored to the specific needs and context of that county. HXI took a very specific bottom-up approach in which counties were required to follow a pre-structured process of selection and adaptation of international best practices [4,12-14]. This somewhat contrasted with the more common definition of bottom-up reform, which refers to developing policy and interventions in ‘a joint process of formulation and implementation involving networks and coalitions’ [9].”*

R2.18 Page 12. Line 326 I thought there were 22 indicators, and yet when I arrive at this point in the paper the Reader is advised there are 166 indicators. I am therefore confused by the lack of clarity on the number of targets etc. Please clarify and report so that the Reader is clear on which targets, set by whom, how many and in which cycle of the process in the results section.

*Reviewer 2 correctly recalls that HXI as 22 indicators, as they are now explicitly provided in S1 Appendix 1, even though the text here mentions that Mei Xian set up a 168 sub indicator performance measurement system, as explained in Appendix 5. These indicators for instance addressed locally prioritized targets such as over prescription of antibiotics which helped to contain cost (HXI indicators 8,9,10,13,…). We hope the clarifications provided in lines 266 onwards which explicitly mention that counties can add indicators serve as an effective first step to reduce the confusion. Moreover, the corresponding text on Mei Xian has now been rewritten as follows:*

*“For example, to reward contribution towards achieving the performance targets, the county Mei Xian needed a set of sub indicators and sub indicator weights to develop a performance-based management system (see Appendix 5, Box 1). The first system design developed by provincial academic experts included 168 sub indicators. However, it appeared too complex to influence management and professional behaviours towards the targeted performance improvement and was difficult to operate as well.”*

R2.19 Page 13, line 366 Please explain in which ways you understand the three counties to have been ‘ready for change’. Readiness for change is a critical part of reform adoption and implementation, thus the Reader would benefit from this information. Please link this to your understanding to readiness for change as defined by Greenhalgh.

*The readiness for change [10,11] of the three case study counties was described in the project report [14] but indeed not in the results section. In choosing the focus of the manuscript and which results to include, we had decided to leave this theme out, which caused some inconsistencies between the discussion and the results section as rightfully pointed out by Reviewer 2 (and perhaps also implied by some of the comments of Reviewer 1). This issue is resolved as there is now a full paragraph early in the results section on the readiness for change of each of the three counties:*

*“The three case study counties welcomed HXI and had already earned recognition for prior achievements. They joined HXI aiming to resolve issues they had already identified prior to starting HXI. Mei Xian, for instance, had earned the distinction of being a ‘national health county’ for its achievements in (public) health services and had participated in HVIII. Within the public health component of HXI, Mei Xian was eager to start performance-based management, which they had already unsuccessfully tried to implement in 2006. Xi Xian was considered to have also performed well in HVIII, during which they developed their first ideas about jointly reforming hospital financing and performance.*

*“HVIII basically didn’t provide a comprehensive intervention. But for the time when we had HXI, it was as if all the pieces were there.“*

*(respondent H1)*

*Despite past performance recognitions, Jiulongpo was initially not selected for HXI. The county lobbied hard to be included, thereby causing their province to have six counties participating in HXI rather than the intended number of five.”*

R2.21 Page 13, line 380. Please list three examples of targets achieved. The reason I ask this is a simliar reason I asked for the table earlier of the 22 indicators, while success is mentioned in the introduction I as the Reader do not have a sense of what success was achieved, I realise that the success/benefits are published in another paper but it would be nice to have some sense within the paper. I realise more information is given in the Appendix. I am not asking for a major change in the paper in any way, just a sense here and there. The goals of the reform are listed once only in the introduction but connections are not made again explicitly to these goals in the paper, linking your findings in some way to these goals more explicitly will help the Reader stay connected to the overall goals.

*We have now stated in the introduction that the World Bank’s evaluation states that all well-specified performance targets on the 22 project indicators were ‘achieved’ or ‘surpassed’ [4]. The indicators specified in S1 Appendix 1, and their description provide detailed information where possible.*

*Moreover, the introduction now mentions examples of implemented interventions (please be aware that overall more than 200 interventions have been implemented):*

*“Examples of selected and adapted interventions for each of the three areas are 1) improving NCMS coverage and integration with MA (to improve rural health financing), 2) introducing new hospital payment systems (e.g., case-based payment) and introducing performance-based payment in hospitals and lower-level facilities, and 3) training public health workers as well as the population on hypertension and diabetes management. See Table 1 of [15] for a comprehensive overview.”*

## Miscellaneous Comments

R2.0	Thank you for the opportunity to review your paper. I found resonance with my own work. The research team set out to explore the implementation of a bottom up process of reform in rural China in a context of a traditionally hierarchical system. A ‘help it happen’ approach was adopted, distinctive in its bottom up approach to reform implementation, as different from a traditional top down approach to implementation.

R2.15 Page 8, line 200 Spelling mistake on ‘implementations’

*Thank you for catching the spelling error. After the revisions we have run a renewed complete spell check and had the article read by a native English proofreader. We hope all spelling mistakes, including the ones you have identified are now resolved.*

R2.16 Page 8, line 218 – 219 I am not surprised by the many iterations and adaptations, this is the nature of bottom up processes in implementation. What is interesting here is that implementers expected things to work out the first time round – this tells me that perhaps there was a lack of communication on what bottom up reform is and that in fact adaptation and learning is what should be expected.

*Thank you for this comment. We hope the text of the results section of the original manuscript did not give you the impression that we were surprised by the iterative process. The text simply intended to reflect that the first implementations delivered considerably below expectations. Obviously, this implied that it was more difficult to achieve the project objectives and created and counties now realized it was their responsibility to adapt and persist. In our view this was not so much do to a lack of communication, but rather a learning process for all involved. After reflection, we have chosen not to make any adjustment to the text in the Results section.*

R2.20 Page 13, line 365 – 374 A suggestion: I assume also based on your results that the reason it was hard in the beginning is because identifying innovations based on international literature is a very hard thing to do, local level actors are often not trained to read empirical literature and then to rank interventions using evidence. I think if you have any knowledge about the process of the experts engaging with the international and sharing with the local actors this would be good to know to. I think it may be unfair to say only 6 out of 40 achieved this on schedule as it is a highly complex process of sorting through technical data. As mentioned, in the introduction it would be good to know what the model of bottom up implementation is – does it include evidence based innovations only or home grown also?

*The introduction now clearly states that the prescribed bottom up approach of HXI has been to select interventions from suggested international best practices and then to adapt and implement – no home grown innovations. This is and was further described in the opening sentences of the Results section:*

*“The counties were subsequently expected to select, adapt, and implement health system innovations (sometimes referred to as interventions below) according to yearly plans developed at county level to address the self-formulated priorities.”*

*We agree with Reviewer 2 that selecting and adapting innovations tailored to local needs is ‘a highly complex process of sorting through technical data’. However, we don’t consider it ‘unfair to say only 6 out of 40 achieved this on schedule’, as these are the facts found by our research and reported without reluctance. Respondents added that even these six counties only succeeded after much unanticipated support. These findings appear to indicate that the initial schedule was overly optimistic. We believe these facts are relevant as they bring out insightful interactions between the top down planning and control approaches practiced and the bottom up innovations processes designed to take place in HXI. We have therefore decided to leave the corresponding reporting of results unchanged.*

R2.22 Things to consider for the Conclusion: please see my comment above linked to page 13, line 365 – 374 and take into consideration if my comment is relevant. I think important to note in the conclusion that while this reform process was labelled as ‘bottom up” it was in fact an interplay between top down and bottom up that drove success, which given the nature of expert support in this context is plausible.

*We have learned a lot from the comments of Reviewer 2, which has led to adjustment of the Introduction and Research Methods, but also to adjustment of the Discussion and Conclusions. In relation to the above, we have now adjusted the Discussion section to reflect upon the interaction between top down and bottom up processes taking place in HXI’s hybrid approach. It specifically details out that the hybrid approach was not simply an ‘in between’ (help it happen) approach, but blended these with top down (make it happen) and bottom up (let it happen) elements:*

*“Taken together, the above findings suggest that the continuum of healthcare reform implementation approaches in which hybrid approaches reside—from bottom-up to top-down—has two dimensions: a content dimension and a procedural dimension. Within the content dimension, HXI relied on bottom-up practices such as diffusion, experimentation, adaption, autonomous and eventually double-loop learning [23,32,35], which are practices that match a ‘let it happen’ paradigm, as seen in the authoritative review of Greenhalgh et al. [10,11] on the spread of health service innovations. To enable bottom-up innovation in the content dimensions, HXI relied on top-down practices in the procedural dimension. All county innovation activities were tightly supervised through an extensive planning and control framework, which included the release of funding. This top-down approach within the procedural dimensions matches a ‘make it happen’ perspective to disseminating innovation 11,36].*

*Between ‘let it happen’ and ‘make it happen’, Greenhalgh et al. [11] position the ‘help it happen’ perspective, which emphasizes negotiation, influencing and enabling. Our findings confirm that such ‘help it happen’ practices played an important role in HXI. Both the HXI leadership itself and the experts intentionally played enabling roles, providing ‘guidance and support’ but ‘no orders’. The relationships with county-level leadership were collaborative, and they were sometimes characterized as being ‘friends’.*

*The role of county-level leaders appears to have been pivotal and essential. While influencing and negotiating with a complex stakeholder network (‘help it happen’), they concurrently had to manage a novel bottom-up process on the content of innovation at the county level (‘let it happen’) and traditional top-down practices from higher levels (‘make it happen’). They needed to handle the full complexity of HXI’s ambitious design, which used much of the top-down to bottom-up continuum. Such a hybrid approach that explicitly blends procedural top-down elements into a bottom-up approach fits the rural Chinese context well but appears not to have been reported previously [10,11,32].”*

R1.9 The analysis of data is weak. The arguments do not sufficiently support the conclusions.

*We hope that with the revised theoretical embedding, research question, and method section, Reviewer 1 no longer believes the analysis of the data to be weak, nor that the arguments do not sufficiently support the conclusions. Especially now the Discussion and Conclusion have been rewritten to more closely relate to the revised research question and methods. The comments of Reviewer 2 and our response to these comments may be further instrumental to change perspective as well.*

R1.10 A table with information (including such as case number/ age/ gender/ organization/ position et al.) of the participants should be presented in the paper.

*Given the small pool from which many of the respondents were sampled, revealing their age, gender, position and organization would compromise the anonymity which was promised to obtain consent. Hence, for this research this information can unfortunately not be provided.*

R1.11 In general, this is a project report rather than a scientific paper. The research design and methodology need to be improved. Meanwhile, the theory and analysis of governance in rural China and diffusion of changes are weak, which also need to be improved.

*Hopefully the revised and improved research question and methods help Reviewer 1 to be of the opinion that the presented research is sufficiently scientific and not a project report. The project report was and is mentioned in the References [14]. The theory of diffusion of changes is based on Greenhalgh’s authorative review [10,11]. We believe this very often cited review is an excellent piece of health services research and far from weak. Other provided theoretical concepts, such as the dimensions of organizational culture [19,20] are also widely accepted. Additionally, the revised manuscript now importantly relies on theoretical concepts provided by Reviewer 2. Taken together with the extensive qualitative analysis, as described in the fully revised methods section, we fully believe in all academic integrity and experience, that the analysis is scientifically robust and leads to insightful and important results. Hopefully Reviewer 1 will come to the same conclusion based on the significant revisions made.*

E1. Please ensure that your manuscript meets PLOS ONE's style requirements, including those for file naming. The PLOS ONE style templates can be found at

 E3. We suggest you thoroughly copyedit your manuscript for language usage, spelling, and grammar. If you do not know anyone who can help you do this, you may wish to consider employing a professional scientific editing service.  

*Please see Rebuttal Appendix 3 for proof of editing services.*

*E4: Upon resubmission, please provide the following:*

- *The name of the colleague or the details of the professional service that edited your manuscript*
- *A copy of your manuscript showing your changes by either highlighting them or using track changes (uploaded as a *supporting information* file)*
- *A clean copy of the edited manuscript (uploaded as the new *manuscript* file)*

*A version with changes highlighted (apart from textual/editing changes) is provided.*

E6. Thank you for stating the following financial disclosure:

Yes, the research was funded by The World Bank, IDF Grant for Capacity Building of Evaluation of China's Health System Reform Pilot (No. TF013943). The originally collected data are owned by the Word Bank and can only be shared by the authors with consent of the World Bank.

E7: Please state what role the funders took in the study.  If the funders had no role, please state: "The funders had no role in study design, data collection and analysis, decision to publish, or preparation of the manuscript."

*This sentence is added to the manuscript at the end of the Methods section (and modified slightly as a result of the editing services.) It is also added to the cover letter.*

 E8. We note that you have indicated that data from this study are available upon request. PLOS only allows data to be available upon request if there are legal or ethical restrictions on sharing data publicly. For more information on unacceptable data access restrictions, please see <http://journals.plos.org/plosone/s/data-availability#loc-unacceptable-data-access-restrictions>.

*We are willing to make any data available on request and to make the thick county descriptions report available via any repository or researchgate as soon as the manuscript is accepted for publication.*

In your revised cover letter, please address the following prompts:

If there are ethical or legal restrictions on sharing a de-identified data set, please explain them in detail (e.g., data contain potentially sensitive information, data are owned by a third-party organization, etc.) and who has imposed them (e.g., an ethics committee). Please also provide contact information for a data access committee, ethics committee, or other institutional body to which data requests may be sent.

*As is contractually agreed, the data are owned by The World Bank and can only be shared with their explicit consent. We have added this information, as well as contact data, in the cover letter. It should be noted however that the extensive project report which contains much more data than represented in the article is publicly available from the authors (e.g. via Researchgate).*

E10. Please include captions for your Supporting Information files at the end of your manuscript, and update any in-text citations to match accordingly. Please see our Supporting Information guidelines for more information: <http://journals.plos.org/plosone/s/supporting-information>.

*Done.*

# Rebuttal Appendix 1

## Editor Comments

E0: The reviewers have provided thorough and detailed comments in an effort to strengthen the manuscript and study reporting. Please pay particular attention to the qualitative methods reporting and the integration of the theoretical framework throughout the paper. I look forward to receiving your revised manuscript.

E1. Please ensure that your manuscript meets PLOS ONE's style requirements, including those for file naming. The PLOS ONE style templates can be found at

E2. When reporting the results of qualitative research, we suggest consulting the COREQ guidelines: <http://intqhc.oxfordjournals.org/content/19/6/349>. In this case, please consider including more information on the number of interviewers, their training and characteristics; how participants were selected; if a pilot study was tested; how data was coded; if bias issues were considered.

 E3. We suggest you thoroughly copyedit your manuscript for language usage, spelling, and grammar. If you do not know anyone who can help you do this, you may wish to consider employing a professional scientific editing service.  

E4: Upon resubmission, please provide the following:

- The name of the colleague or the details of the professional service that edited your manuscript
- A copy of your manuscript showing your changes by either highlighting them or using track changes (uploaded as a *supporting information* file)
- A clean copy of the edited manuscript (uploaded as the new *manuscript* file)

E5. Please provide additional details regarding participant consent. In the ethics statement in the Methods and online submission information, please ensure that you have specified (1) whether consent was informed and (2) what type you obtained (for instance, written or verbal). If your study included minors, state whether you obtained consent from parents or guardians. If the need for consent was waived by the ethics committee, please include this information.

 E6. Thank you for stating the following financial disclosure:

Yes, the research was funded by The World Bank, IDF Grant for Capacity Building of Evaluation of China's Health System Reform Pilot (No. TF013943). The originally collected data are owned by the Word Bank and can only be shared by the authors with consent of the World Bank.

E7: Please state what role the funders took in the study.  If the funders had no role, please state: "The funders had no role in study design, data collection and analysis, decision to publish, or preparation of the manuscript."

If this statement is not correct you must amend it as needed.

Please include this amended Role of Funder statement in your cover letter; we will change the online submission form on your behalf.

 E8. We note that you have indicated that data from this study are available upon request. PLOS only allows data to be available upon request if there are legal or ethical restrictions on sharing data publicly. For more information on unacceptable data access restrictions, please see <http://journals.plos.org/plosone/s/data-availability#loc-unacceptable-data-access-restrictions>.

In your revised cover letter, please address the following prompts:

a) If there are ethical or legal restrictions on sharing a de-identified data set, please explain them in detail (e.g., data contain potentially sensitive information, data are owned by a third-party organization, etc.) and who has imposed them (e.g., an ethics committee). Please also provide contact information for a data access committee, ethics committee, or other institutional body to which data requests may be sent.

b) If there are no restrictions, please upload the minimal anonymized data set necessary to replicate your study findings as either Supporting Information files or to a stable, public repository and provide us with the relevant URLs, DOIs, or accession numbers. For a list of acceptable repositories, please see <http://journals.plos.org/plosone/s/data-availability#loc-recommended-repositories>.

We will update your Data Availability statement on your behalf to reflect the information you provide.

E9. Your ethics statement must appear in the Methods section of your manuscript. If your ethics statement is written in any section besides the Methods, please move it to the Methods section and delete it from any other section. Please also ensure that your ethics statement is included in your manuscript, as the ethics section of your online submission will not be published alongside your manuscript.

E10. Please include captions for your Supporting Information files at the end of your manuscript, and update any in-text citations to match accordingly. Please see our Supporting Information guidelines for more information: <http://journals.plos.org/plosone/s/supporting-information>.

## Reviewer 1 Comments:

R1.1. Background: please give more details of the intervention package of Health XI project in order to help the audience to better understand what the role and importance of "help it happen" approach in the project.

R1.2. Research question: even though current literature showed the effectiveness of Health XI project somehow in China, it does not mean "bottom-up" reform happened in China. There might be many other factors/interventions contributing to the positive result of the Health XI project.

R1.3 Sampling strategy: the criteria for selection of research participants is not clear. Meanwhile, the authors did not say how to achieve information saturation. It seems the sampling is random. A minor question: in the text, it says 3 group interviews were conducted (Line 105). But in Table 1, there shows 4 focus group discussions.

R1.4 Interviewers: because qualitative researchers themselves are research tools, it is important to state in detail who did the interviews: do they have any experience in qualitative study? Have they both been trained for this study? How did the two interviewers speaking different language work together? Also, who did data analysis? How did the researchers do data analysis?

R1.5 Working language: it is very confusing. Regarding interviews, “these interviews were conducted in Chinese using certified interpreters who interpreted consecutively” (line 122-123). Does it mean the Chinese interviewer did the interviews in Chinese, and a translator did translation during the interview? Or how did you organize the interviews? It is not clear in the manuscript. Regarding data analysis, “the English texts of the interviews were transcribed using audio files of the original and translated interview content” (line 150-151). If the interviews are bilingual, how were they been transcribed and translated? This should be described more clearly in the paper.

R1.6 Ethics: because the study involved human participants, please list the name of the IRB or ethics committee and other information of the ethical approval. Otherwise, you should give sufficient information for why not obtaining ethical approval.

R1.7 The trustworthiness of the results has been affected by the vagueness of the research method.

R1.8 The authors used a conceptual model of innovation in the analysis. But as a qualitative research, the original model should be deduced, deepened and finalized into a new model.

R1.9 The analysis of data is weak. The arguments do not sufficiently support the conclusions.

R1.10 A table with information (including such as case number/ age/ gender/ organization/ position et al.) of the participants should be presented in the paper.

R1.11 In general, this is a project report rather than a scientific paper. The research design and methodology need to be improved. Meanwhile, the theory and analysis of governance in rural China and diffusion of changes are weak, which also need to be improved.

## Reviewer 2 Comments

R2.0	Thank you for the opportunity to review your paper. I found resonance with my own work. The research team set out to explore the implementation of a bottom up process of reform in rural China in a context of a traditionally hierarchical system. A ‘help it happen’ approach was adopted, distinctive in its bottom up approach to reform implementation, as different from a traditional top down approach to implementation.

R2.1 Page 3, line 42 – 43 Please include what these health system reforms hope to achieve. Please add at least five words describing the overarching goal of health systems reform in China is. ….. “a series of national health system reforms *to achieve XXXXX* and achieved considerable improvements in areas such as XXXXXXX (1.2}

R2.2 Other comments on introduction: The introduction primarily speaks to literature on China, please provide a definition of ‘bottom up approach’ with some international empirical examples of what typically happens in a bottom up approaches to implementation, you might also want to clarify for the reader what you mean by top down implementation. This will help the Reader to situate the work in broader literature on bottom up implementation and why this work is unique.

R2.3 Not all readers will know what top down reform/implementation versus bottom up reform/implementation is so it would be good to situate the work – in all implementation of policies bottom up processes happen as implementers inevitably use their discretion in implementation, the difference in your study is that bottom up processes were intentional. Perhaps just one paragraph with a few key definitions and some empirical conclusions on bottom up innovations in other contexts. You could add this to paragraph two.

R2.4 Please define what ‘help it happen approach’ is. What are the key steps or principles? In your context it seems that there are pre-set targets and there is a requirement that evidence-based innovations are to be used when counties select innovations? Also a huge expert presence in this model. This bottom up model is unique and the Reader should know this. The authors do note it is based on the principle of ‘adaptation’ but it would be good to know a bit more about this process specifically.

R2.5 Page 4, line 85 I struggled with the fact that the research question was a ‘what’ question - this is typically a quantitative style of research question. This research, being qualitative in nature was more exploratory trying to identify how and why success was achieved. I suggest rephrasing the question to: *How and in what contextual conditions did a bottom up reform process achieve success in three rural Chinese districts?* This reflects a more appropriate exploratory study which this is, also given the importance of understanding context when presenting qualitative work.

R2.6 Page 4, line 90 Please make clear in the first paragraph to the Reader what the unit of analysis is in this paper. Are you primarily exploring the routinisation and adoption of a bottom up process of designing local innovations? Or are you primarily exploring the implementation of health reforms? Please link back to you research question. The process of trying to introduce bottom up processes (in a context of typically top down hierarchy) is different from implementing reforms, even though they are linked. Good to also make link clear.

R2.7 Page 4, line 97 Please explicitly include the selection criteria for the three districts. How and why were these three selected from all the best practise counties.

R2.8 Page 6, line 136 - 144 The application of theory and the use of conceptual frameworks is what allows qualitative research to be transferable to other contexts, there is currently too little information on the key concepts and theory applied. Please provide more detail on why the Greenhalgh f/work was used and what specifically concepts were used in this study, please define the key concepts.

R2.9 Please explain why the model by the World Bank was used and why you felt it relevant to merge the two (merging is fine but the Reader should know why this specific two were merged). Please provide an explanation of what is meant by “enriched with topics from organisational culture and learning”. What do these concepts mean and how is it relevant to be used in this paper. While these concepts may be intuitive to some from the social sciences, the PLOS audience goes beyond the social sciences. Also given that these concepts are used in analysis this will further support deep understanding of the relevance of the work.

R2.10 In Appendix 4 a topic listing of words is insufficient, please include a definition of each of the concepts alongside the word. The Reader should be able to see a definition of each concept while they are reading to help understand the content.

R2.11Page 7, line 167 Please input a table that lists the 22 indicators, I felt as though I needed to know what this was to fully understand the intentions of the reform. Tables typically don’t add to word count so this shouldn’t be written in paragraph form but rather in a Table.

R2.12 Page 7, 169 I am confused by the statement ‘self -formulated priorities’, given that they were asked to achieve a specific set of 22 indicators which means that the targets/priorities were already set by national. I think that what you are trying to say is that they were given the authority to develop bottom up interventions to achieve the pre set targets? If you could clarify that would be good. You also state in the next paragraph ‘prioritised improvement targets’.

Then on page 9, line 223 the authors write “self-set improvement targets”. Is this a selection from the 22 target indicators?

R2.13 Only on page 10, line 256 is the Reader made aware that the counties could add targets. I am now confused as to whether the targets/priorities spoken about earlier in the results section are referring to these new targets or the old targets.

R2.14 Page 7, line 183 Could you specify if counties were expected to only develop evidence-based interventions using international best practice? Or were they also allowed to design innovations based on an understanding of their own context and to think outside of the [evidence-based] box? This is an important point to understand the nature of bottom up innovation in this paper. One understanding of bottom up processes is where implementers craft innovations from their own understandings of context and experiences. Your paper seems to include a particular nuance on top of this.

R2.15 Page 8, line 200 Spelling mistake on ‘implementations’

R2.16 Page 8, line 218 – 219 I am not surprised by the many iterations and adaptations, this is the nature of bottom up processes in implementation. What is interesting here is that implementers expected things to work out the first time round – this tells me that perhaps there was a lack of communication on what bottom up reform is and that in fact adaptation and learning is what should be expected.

R2.17 Page 10 Suggestion: I really like that there is recognition in the paper that this model is in fact an interplay between top down and bottom up reform/implementation. It might be useful for the authors to acknowledge in the introduction that this model looks like a hybrid of top down / bottom up rather than fully bottom up. The authors do not have to do this, but it might be good to qualify this upfront. I think this links to me earlier point in the introduction section where the work needs to be situated briefly in theory on what top down implementation is versus bottom up and the inevitability of both being present.

R2.18 Page 12. Line 326 I thought there were 22 indicators, and yet when I arrive at this point in the paper the Reader is advised there are 166 indicators. I am therefore confused by the lack of clarity on the number of targets etc. Please clarify and report so that the Reader is clear on which targets, set by whom, how many and in which cycle of the process in the results section.

R2.19 Page 13, line 366 Please explain in which ways you understand the three counties to have been ‘ready for change’. Readiness for change is a critical part of reform adoption and implementation, thus the Reader would benefit from this information. Please link this to your understanding to readiness for change as defined by Greenhalgh.

R2.20 Page 13, line 365 – 374 A suggestion: I assume also based on your results that the reason it was hard in the beginning is because identifying innovations based on international literature is a very hard thing to do, local level actors are often not trained to read empirical literature and then to rank interventions using evidence. I think if you have any knowledge about the process of the experts engaging with the international and sharing with the local actors this would be good to know to. I think it may be unfair to say only 6 out of 40 achieved this on schedule as it is a highly complex process of sorting through technical data. As mentioned, in the introduction it would be good to know what the model of bottom up implementation is – does it include evidence based innovations only or home grown also?

R2.21 Page 13, line 380 Please list three examples of targets achieved. The reason I ask this is a simliar reason I asked for the table earlier of the 22 indicators, while success is mentioned in the introduction I as the Reader do not have a sense of what success was achieved, I realise that the success/benefits are published in another paper but it would be nice to have some sense within the paper. I realise more information is given in the Appendix. I am not asking for a major change in the paper in any way, just a sense here and there. The goals of the reform are listed once only in the introduction but connections are not made again explicitly to these goals in the paper, linking your findings in some way to these goals more explicitly will help the Reader stay connected to the overall goals.

R2.22 Things to consider for the Conclusion: please see my comment above linked to page 13, line 365 – 374 and take into consideration if my comment is relevant. I think important to note in the conclusion that while this reform process was labelled as ‘bottom up” it was in fact an interplay between top down and bottom up that drove success, which given the nature of expert support in this context is plausible.

## Rebuttal Appendix 2: Qualitative studies by the interviewers (authors)

1. Yuan S, Wang F, Li X*, et al* Facilitators and barriers to implement the family doctor contracting services in China: findings from a qualitative study, *BMJ Open,* 2019;**9:**e032444.
2. [Outcomes in patients with chronic uveitis: which factors matter to patients? A qualitative study](javascript:void(0)) AC Stolk-Vos, H Kasigar, KJ Nijmeijer, TO Missotten, JJ Busschbach, Joris van de Klundert, Leoniek van W Kranenburg, BMC ophthalmology 20 (1), 1-8
3. Alingh, C.W., van Wijngaarden, J.D.H., Huijsman, R. *et al.* The influence of environmental conditions on safety management in hospitals: a qualitative study. *BMC Health Serv Res* **18,** 313 (2018).
4. van Ineveld, M, van Wijngaarden, J, Scholten, G. Choosing cooperation over competition; hospital strategies in response to selective contracting. *Int J Health Plann Mgmt*. 2018; 33: 1082– 1092.
5. Weggelaar-Jansen, A.M., van Wijngaarden, J. Transferring skills in quality collaboratives focused on improving patient logistics. *BMC Health Serv Res* **18,** 224 (2018).
6. den Herder-van der Eerden, M., van Wijngaarden, J., Payne, S., Preston, N., Linge-Dahl, L., Radbruch, L., … Hasselaar, J. (2018). Integrated palliative care is about professional networking rather than standardisation of care: A qualitative study with healthcare professionals in 19 integrated palliative care initiatives in five European countries. *Palliative Medicine*, *32*(6), 1091–1102, 2018.
7. The Role of a Multicomponent Home-Health Intervention in Reducing Caregiver Stress in Singapore: A Qualitative Study, [Michael J Penkunas](javascript:;), [Angelique W M Chan](javascript:;), [Chek Hooi Wong](javascript:;), [Dirk F de Korne](javascript:;), [See Mieng Tan](javascript:;), [Sweet Fun Wong](javascript:;), The Journals of Gerontology: Series B, Volume 73, Issue 2, 1 February 2018, Pages 314–325,
8. Stolk-Vos, A. C., van der Steen, J. J. E., Drossaert, C. H. C., Braakman-Jansen, A., Zijlmans, B. LM., Kranenburg, L. W., & De Korne, D. F. (2018). A Digital Patient-Led Hospital Checklist for Enhancing Safety in Cataract Surgery: Qualitative Study. JMIR Perioperative Medicine, 1(2),
9. Why patients prefer high-level healthcare facilities: a qualitative study using focus groups in rural and urban China, Yun Liu Y, Liwei Zhong L, Shasha Yuan S, Joris van de Klundert, BMJ Global Health, 2018; **3:**e000854. (Q1)
10. We are planning to leave, all of us - A qualitative study into the mechanisms explaining healthcare employee turnover in rural Ethiopia, Judith van Dongen- van den Broek, Ebrahim Mohammed Yesuf, Jasmijn Vreugdenhil, Saeid Mohammed Yimer, Joris van de Klundert, Human Resource for Health, 2018, 16:37. (Q1)
11. Service quality of primary healthcare institutions under the vertically integrated model: A perspective of demanding sideTop of Form
12. Bottom of Form
13. , Shasha YUAN; Zhipeng YONG; Fang WANG; Jun ZHAO; Xi LI; Yongchao CHEN; Hongyi DU, [Chinese Journal of Health Policy](http://www.yxxxx.ac.cn/)  2017;10(7):41-4
14. de Vos, A., Cramm, J.‐M., van Wijngaarden, J. D. H., Bakker, T. J. E. M., Mackenbach, J. P., and Nieboer, A. P. ( 2017) Understanding implementation of comprehensive geriatric care programs: a multiple perspective approach is preferred. *Int J Health Plann Mgmt*, 32: 608– 636
15. Ewert B, Hodiamont F, van Wijngaarden J*, et al,* Building a taxonomy of integrated palliative care initiatives: results from a focus group, *BMJ Supportive & Palliative Care* 2016;6:14-20.
16. [Can relational coordination help inter-organizational networks overcome challenges to coordination in patient portals?](https://scholar.google.com/citations?view_op=view_citation&hl=en&user=RleRiacAAAAJ&sortby=pubdate&citation_for_view=RleRiacAAAAJ:oldoQiaHq2UC), T Otte-Trojel, TG Rundall, A de Bont, J van de Klundert, International Journal of Healthcare Management, 1-9, 2016, Q2
17. [The organizational dynamics enabling patient portal impacts upon organizational performance and patient health: a qualitative study of Kaiser Permanente](https://scholar.google.com/citations?view_op=view_citation&hl=en&user=RleRiacAAAAJ&sortby=pubdate&citation_for_view=RleRiacAAAAJ:i91s68tWr-MC), T Otte-Trojel, TG Rundall, A de Bont, J van de Klundert, ME Reed, BMC health services research 15 (1), 1, 2016, Q1
18. [Developing patient portals in a fragmented healthcare system](https://scholar.google.com/citations?view_op=view_citation&hl=en&user=RleRiacAAAAJ&sortby=pubdate&citation_for_view=RleRiacAAAAJ:M0j1y4EgrScC),T Otte-Trojel, A de Bont, M Aspria, S Adams, TG Rundall, J van de Klundert, International Journal of Medical Informatics 84 (10), 835-846, 2015, Q1
19. Weggelaar-Jansen, A.M., van Wijngaarden, J. & Slaghuis, S. Do quality improvement collaboratives’ educational components match the dominant learning style preferences of the participants?. *BMC Health Serv Res* 15, 239 (2015).

# Rebuttal Appendix 3: Editing Certificate
