# Supplementary material for: ‘Hybrid’ top down bottom up health system innovation in rural China: A qualitative analysis
Source: PLoS One. 2020 Oct 7;15(10):e0239307. doi: 10.1371/journal.pone.0239307 (PMC7540887; doi:10.1371/journal.pone.0239307)
Supplement: Attachment — Submitted filename: From Reviewer 2 PONE-D-19-27227 PLOS round 2 20062020.docx [file pone.0239307.s009.docx]

From Reviewer 2 (second round of comments)

Manuscript: PONE-D-19-27227R1

Introduction, page 3, line 48: Are all 800 million citizens in China rural? If not, please remove the word rural, it is confusing later when ‘rural’ is spoken about as a sample of the population rather than the whole population.

Line 67: IS HXI the acronym for China’s Rural Health Reform Project Health XI? If yes, please put in brackets at first use after the full word.

Line 90: Table 1 is referenced in this line as examples of interventions, but the information provided in Table 1 on page 7 does not match. Please correct.

Appendix 1: there is a spelling error in “The 22 Monitoring and Evaluation Indications of HXI”

Line 168: which ministry?

Line 172-174: I think there is a mix up with the Appendix numbering, the interview protocol is currently Appendix 5, not Appendix 4. Please check numbering of all appendices and correct where necessary.

Line 206-214: The bullet point style of numbering in this section looks out of place, perhaps write as follows: “These interviews were conducted as follows; (1) the researchers’ questions were always asked in English; (2) a certified interpreter translated the questions into Chinese and then asked them to the respondents; (3) …..

Line 241-257: In the data analysis section please provide at minimum one methodological reference that you consulted to develop your process of coding and analysis etc.

Line 251: you may want to also reference again the conceptual literature used (refs 19-23?) as you refer to these concepts again here (I realise it has been referenced earlier but it would be proper to reference again here as the concepts explicitly drawn on in the sentence.

Line 286: What is HVIII? This is a new acronym that is introduced but there is no full word description.

The discussion section: It is much improved, I value the analysis in the context of the Greenhalgh frameworks. But now that I can see the full body of work and can understand the research question in full, I have a suggestion (this is a suggestion) – you are meant to locate the discussion section in the context of other empirical literature, there are no other empirical examples referenced in your discussion section other than the work by Greenhalgh – you could identify other papers that apply the Greenhalgh concepts or that discuss a hybrid approach and compare/contrast with your findings to enrich your discussion section.
