# Supplementary material for: ‘Hybrid’ top down bottom up health system innovation in rural China: A qualitative analysis
Source: PLoS One. 2020 Oct 7;15(10):e0239307. doi: 10.1371/journal.pone.0239307 (PMC7540887; doi:10.1371/journal.pone.0239307)
Supplement: Attachment — Submitted filename: second revision letter.docx [file pone.0239307.s010.docx]

***Response to second round editor and reviewer report for the revision of PONE-D-19-27227R1 ‘Hybrid’ Top down Bottom up Health System Innovation in Rural China: a qualitative analysis***

We are very grateful to the editor and second reviewer for handling our revision so carefully and timely in the challenging dynamics of the on-going COVID19 dynamic and wish both of you continue to be safe and well!

Below we respond to the suggestions and comments one by one, aiming to completely resolve each one of them, resulting in a version of the manuscript that can be accepted for publication.

*Thank you for your thorough response to the reviewers' comments, particularly in light of current global circumstances. We feel the manuscript is much strengthened, but have a few outstanding comments. Please address each of the reviewers' comments and the below. The reviewer has made a suggestion regarding the Discussion - I would appreciate your response or brief consideration of the literature.*

- Thank you for appreciation, we too believe the manuscript has benefitted substantially from the previous revision and remain grateful for the thoughtful feedback and suggestions made in the first review round. Hopefully, you find the second round comments and the suggestion are completely and satisfactorily addressed in the revised version and as indicated pointwise below.

*The data availability and funding disclosure statements seem to be conflicting. In your statement about the involvement of the funder, please add that "the external evaluation of HXI was commissioned by the World Bank" and explain the relationship to data ownership.*

- The suggested phrase "the external evaluation of HXI was commissioned by the World Bank" has been added in the revised manuscript under Financial Disclosure (lines 566), and in the statements made during manuscript submission, as well as the cover letter.

*Minor comments:*

*- Abstract: Please address the use of the acronym Project HXI and spell out the first time. Please check the abstract for typos and grammar, including "hybrid'" (line 23), and several sentence fragments (lines 26-29)*

🡪 Thank you for these suggestions. The abstract has been checked for typos and grammar and has been adjusted as suggested. The sentences on lines 26-29 have been revised.

*- Introduction: be consistent in the use of double vs single quotation marks; line 106 and 108, what is "a large power difference?"*

For consistency, we have removed the occurrences of double quotation marks. Power distance is a relatively well-known dimension of organizational culture, as elaborated in references [19] and [20]. We have now put it between single quotation marks to make explicit this construct is taken from the mentioned references, rather than a common sense expression. Thank you for pointing out that not every reader may be familiar with the construct and literature and helping us to improve the manuscript accordingly.

*If you would like to make changes to your financial disclosure, please include your updated statement in your cover letter. Guidelines for resubmitting your figure files are available below the reviewer comments at the end of this letter.*

We have made statements regarding financial disclosure in the cover letter as well.

*Reviewer #2: Introduction, page 3, line 48: Are all 800 million citizens in China rural? If not, please remove the word rural, it is confusing later when ‘rural’ is spoken about as a sample of the population rather than the whole population.*

- We thank the reviewer for pointing out to us that the previous formulation of the sentence may cause misunderstanding. The population of China is around 1.3 billion (1300 million) of whom 800 million were classified as rural in 2009. Hence, the answer to the reviewer’s first question is negative as it misrepresents China’s population size. The subsequent suggestion made by the reviewer is invalid for the same reason. We have instead adjusted the text as follows, to avoid any misunderstandings about the size of China’s population:

“These reforms have been further strengthened by additional health policy measures specifically targeted at China’s 800 million rural citizens, who formed more than half of China’s population in 2009 and comprised close to 10% of the global population [4-8].”

*Line 67: IS HXI the acronym for China’s Rural Health Reform Project Health XI? If yes, please put in brackets at first use after the full word.*

- Thank you, we have modified the manuscript accordingly.

*Line 90: Table 1 is referenced in this line as examples of interventions, but the information provided in Table 1 on page 7 does not match. Please correct.*

- The text refers to Table 1 of reference [15] not to Table 1 of the manuscript itself. We believe the best solution is to leave the text unchanged.

*Appendix 1: there is a spelling error in “The 22 Monitoring and Evaluation Indications of HXI”*

- Thank you, we have fixed the spelling error.

*Line 168: which ministry?*

- Thank you, we have now clarified the ministry (in fact the National Health & Family Planning Commission).

*Line 172-174: I think there is a mix up with the Appendix numbering, the interview protocol is currently Appendix 5, not Appendix 4. Please check numbering of all appendices and correct where necessary.*

- Thank you for catching this one, there was indeed a mix up in the files names of Appendices 4 and 5. We have verified all appendix references, and appendix file names as suggested and they are correctly referenced now.

*Line 206-214: The bullet point style of numbering in this section looks out of place, perhaps write as follows: “These interviews were conducted as follows; (1) the researchers’ questions were always asked in English; (2) a certified interpreter translated the questions into Chinese and then asked them to the respondents; (3) …..*

- Thank you, we have modified the manuscript accordingly.

*Line 241-257: In the data analysis section please provide at minimum one methodological reference that you consulted to develop your process of coding and analysis etc.*

- Thank you, we have modified the manuscript accordingly, by repeating the methodological references provided in the introduction of the methods section.

*Line 251: you may want to also reference again the conceptual literature used (refs 19-23?) as you refer to these concepts again here (I realise it has been referenced earlier but it would be proper to reference again here as the concepts explicitly drawn on in the sentence.*

- Thank you, we have modified the manuscript accordingly.

*Line 286: What is HVIII? This is a new acronym that is introduced but there is no full word description.*

- Thank you for pointing out that the reader has indeed never been introduced to HVIII, as the manuscript implicitly assumed. Project HVIII (‘health 8’) was well known to all respondents as a predecessor project sponsored by the World Bank to improve rural Health in China. Without going into unnecessary detail, the revised manuscript provides a brief explanation: “…and had participated in a predecessor World Bank sponsored health sector reform project called Health VIII (HVIII)..”

*The discussion section: It is much improved, I value the analysis in the context of the Greenhalgh frameworks. But now that I can see the full body of work and can understand the research question in full, I have a suggestion (this is a suggestion) – you are meant to locate the discussion section in the context of other empirical literature, there are no other empirical examples referenced in your discussion section other than the work by Greenhalgh – you could identify other papers that apply the Greenhalgh concepts or that discuss a hybrid approach and compare/contrast with your findings to enrich your discussion section*

- We have added a parapraph (lines 532-541) to elaborate the position of the findings in related empirical literature on hybrid health reforms. In the concluding sentences we compare and contrast with the presented findings. Thank you for this suggestion, it has enriched the discussion.
- After rereading the manuscript and working on the discussion, we have restructured part of the introduction (which actually had a repeating sentence). We believe the revised part (lines 62-77) forms an additional improvement resulting from this second round of revision.
